# Discovering Novel *Alternaria solani* Succinate Dehydrogenase Inhibitors by *in Silico* Modeling and Virtual Screening Strategies to Combat Early Blight

**DOI:** 10.3389/fchem.2017.00100

**Published:** 2017-11-17

**Authors:** Sehrish Iftikhar, Ahmad A. Shahid, Sobia A. Halim, Pieter J. Wolters, Vivianne G. A. A. Vleeshouwers, Ajmal Khan, Ahmed Al-Harrasi, Shahbaz Ahmad

**Affiliations:** ^1^Institute of Agricultural Sciences, University of the Punjab, Lahore, Pakistan; ^2^Center of Excellence in Molecular Biology, University of the Punjab, Lahore, Pakistan; ^3^Department of Biochemistry, Kinnaird College for Women, Lahore, Pakistan; ^4^Plant Breeding, Wageningen University and Research, Wageningen, Netherlands; ^5^Department of Chemistry, COMSATS Institute of Information Technology, Abbottabad, Pakistan; ^6^UoN Chair of Oman Medicinal Plants and Marine Products, University of Nizwa, Nizwa, Oman

**Keywords:** *Alternaria solani*, succinate dehydrogenase, homology modeling, pharmacophore modeling, docking and virtual screening

## Abstract

*Alternaria* blight is an important foliage disease caused by *Alternaria solani*. The enzyme Succinate dehydrogenase (SDH) is a potential drug target because of its role in tricarboxylic acid cycle. Hence targeting *Alternaria solani* SDH enzyme could be efficient tool to design novel fungicides against *A. solani*. We employed computational methodologies to design new SDH inhibitors using homology modeling; pharmacophore modeling and structure based virtual screening. The three dimensional SDH model showed good stereo-chemical and structural properties. Based on virtual screening results twelve commercially available compounds were purchased and tested *in vitro* and *in vivo*. The compounds were found to inhibit mycelial growth of *A. solani*. Moreover *in vitro* trials showed that inhibitory effects were enhanced with increase in concentrations. Similarly increased disease control was observed in pre-treated potato tubers. Hence the applied *in silico* strategy led us to identify novel fungicides.

## Introduction

Potato (*Solanum tuberosum* L.) is a starchy and most widely grown tuberous crop from the *Solanaceae* family worldwide (Brown, 2005; Khorasani et al., 2008; Zaheer and Akhtar, 2016). In Pakistan, potato is an important crop; however its annual production is very low as compared to other countries. Several factors including bacteria, nematode (Gondal et al., 2012), fungus (Ashraf et al., 2012; Mehboob et al., 2013) and virus (Abbas et al., 2012; Gul et al., 2013) along with abiotic factors contributes to the low production of potato in Pakistan. More than eighteen potato diseases have been reported in Pakistan, of which thirteen are very common including black scurf, early and late blights, common and powdery scabs, stem, soft and brown rots, and wilts (Ahmad and Beg, 2001).

Early blight (EB), also known as *Alternaria* blight is distributed worldwide which is a devastating foliage disease, caused by *Alternaria solani* (Ellis & Mart) [Jones and Grout]. EB of potato is the most destructive disease of field crops (Van der Waals et al., 2001), and is significantly common in the USA, Asia and Africa. It develops most rapidly after tuber initiation, it destroy foliage and reduces yield typically by ~20%. However, it is also reported to reduce 70–80% yield in some cases (Bambawale and Bedi, 1982; Stevenson et al., 2001; Olanya et al., 2009; Leiminger and Hausladen, 2012). Since the last few years, EB has been occurring almost every year in Pakistan primarily due to the soil-borne fungal survival, local over-wintering/over-summering of inoculums, cultivation of susceptible varieties and favorable environmental conditions. The disease appears as necrotic lesions of plant leaves. The lesions cause defoliation and reduce the yield (Nachmias et al., 1988). According to Secor and Gudmestad (1999) “It (*A. solani*) almost always affects only the foliar parts of the plant, but can affect tubers and cause a shallow dry rot.” EB is usually controlled by multiple and frequent application of fungicides that showed only moderate effectiveness against EB (Gent and Schwartz, 2003; Pasche et al., 2004; Stevenson et al., 2007; Rosenzweig et al., 2008; Horsfield et al., 2010). The excessive use of pesticides leads to fungicide resistance in *Alternaria* species (Christ and Maczuga, 1989; Holm et al., 2003; Pasche et al., 2004; Miles et al., 2014). *A. solani* is considered as “high-risk” pathogen because of pesticides resistance due to its high genetic variability, abundant sporulation, and polycyclic nature (Van der Waals et al., 2003, 2004; Pasche et al., 2004; Rosenzweig et al., 2008). Respiration inhibitors are the most important class of fungicides in the last 20 years. Resistance to Succinate Dehydrogenase Inhibitors (SDHI's) has become a common phenomenon in many other pathogens (Avenot and Michailides, 2010; Ishii et al., 2011; Avenot et al., 2012). Various fungicides has been developed to control EB, however resistance against these fungicides in *Alternaria* strains (Fairchild et al., 2012) make it an advantageous and interesting task to discover more potent and effective compounds against *Alternaria* to prevent this disease.

SDH catalyzes the oxidation of succinate to fumarate which is a crucial step in the mitochondrial tri-carboxylic acid (TCA) cycle. SDH couples the oxidation of succinate to fumarate with the reduction of ubiquinone to ubiquinol. The two substrates of SDH are present in different mitochondrial compartments; succinate and fumarate are TCA metabolites found in mitochondrial matrix, whereas ubiquinone and its reduced form ubiquinol are hydrophobic electron carriers of the respiratory chain located in the internal mitochondrial membrane. SDH is the only enzyme involved in TCA and electron transport chain (ETC) because it transfer the electrons derived in TCA to the ETC and is considered as an ETC component (Complex II) (Oyedotun and Lemire, 2004; Horsefield et al., 2006).

SDH is composed of four subunits and spatially separated substrate binding sites: SDHA and SDHB that form soluble catalytic dimer which face matrix whereas SDHC and SDHD form cytochrome b membrane spanning anchor (Lemire and Oyedotun, 2002). SDHA is a flavoprotein (Fp) which has succinate binding and oxidation site (Huang et al., 2006), SDHB is an iron sulfur (Ip) cluster protein which is involved in two-step electron transfer from reduced flavin adenine dinucleotide (FAD) to ubiquinone (Cheng et al., 2006). SDHC and SDHD carry a prosthetic b-type heme which might also have a role in the electron transfer to ubiquinone as a cofactor stabilizing the ubiquinone semi-radical formed during the course of this reaction (Anderson et al., 2005). Ubiquinone reduction is a complex process that is not yet fully understood, that occurs at the ubiquinone binding site (Qp site) which is structurally defined by the interface between the SDHB, SDHC, and SDHD subunits (Yankovskaya et al., 2003; Sun et al., 2005; Horsefield et al., 2006; Huang et al., 2006). SDH inhibitors (SDHIs) acts *via* Qp site which is a hydrophobic pocket created by SDHB, SDHC, and SDHD and is highly conserved throughout a range of organisms (Horsefield et al., 2004).

Developing new fungicides is a challenging and time-consuming task. Computational drug designing is a multi-disciplinary field, widely used to find new drug candidates (Abagyan and Totrov, 2001; Lyne, 2002; Schneider and Böhm, 2002). Inhibitors of the mitochondrial respiratory chain enzyme have been developed as antimicrobial agents to control plant pathogenic fungi (Fisher and Meunier, 2007). Drugs that target SDH enzymes could be efficient tools to control pathogens. Virtual screening (VS) is an *in silico* technique used to discover new lead compounds against any specific biological target. VS is used to screen huge numbers of compounds that complement targets of known structure, and eventually select the best predicted compounds to be tested experimentally. With our interest in computational analysis of several biologically important drug targets (Halim et al., 2013, 2015; Halim and Jawad, 2015; Halim and Ul-Haq, 2015; Ul- Haq et al., 2015), we conducted this study to identify novel SDHIs *via in silico* VS. Hence three step screening was applied. Initially, homology modeling was performed to construct the three dimensional (3D) structure of *A. solani* SDH, followed by pharmacophore modeling to screen compounds from the ZINC database. Subsequently, structure based virtual screening (SBVS) was conducted. The compounds that matched with the pharmacophore model were subjected to the SBVS protocol to identify potent inhibitors against *A solani* SDH. The computational strategy is depicted in Supplementary Figure S1. The whole strategy resulted in the identification of twelve potent hits against *A. solani* SDH.

## Materials and methods

### Homology modeling

Initially, 3D structural model of *A. solani* SDH was developed by homology modeling. Modeling was performed by Modeler 9v13 (Fiser and Šali, 2003). The protein sequences of SDHA, SDHB, SDHC and SDHD (SDHA; GenBank: AGS56260.1, GenBank: AGS56262.1, GenBank: AGS56264.1 respectively) were retrieved from the NCBI database (http://www.ncbi.nlm.nih.gov/). The sequences are given in supporting information. After BLAST searching (BLASTp) the templates were selected from the Protein Data Bank (PDB). The sequence identities between templates and *A. solani* SDH subunits are tabulated in Supplementary Table S1. The SDHA sequence was submitted to the automated comparative protein modeling server (http://swissmodel.expasy.org). Based on % identity, SDHB was modeled by templates PDB ID: 1YQ3, 3ABV, 1ZOY, 3VR8, and 1NEK due to high % identities of the flavoprotein and thereonine-sulfur subunits (Supplementary Table S1; Kramer and Cohen, 2004). SDHC and SDHD were also initially modeled by Modeler9v13, however the quality of the obtained SDHC and SDHD models were not good with acceptable stereochemical properties, hence SDHC and SDHD were modeled by I-TASSER. The individual sub-models (SDHA, B, C and D models) were then assembled by superimposing on 1ZOY and the cofactors FAD, FES, SF4, F3S, UQ1, and HEM were transferred inside the defined pockets *via* manual docking using CHIMERA software (Pettersen et al., 2004). Hydrogen atoms were added to the model and energy-minimized by MOE (Molecular Operating Environment, 2017). The multiple sequence alignments of all subunits are depicted in Supplementary Figure S2.

### Model evaluation

The stereochemical properties of the developed model were assessed by PROCHECK (Laskowski et al., 1993), ERRAT (Colovos and Yeates, 1993), and VERIFY-3D (Eisenberg et al., 1997). The results are shown in Supplementary Figures S3, S4 in the supporting information.

### Docking of fungicides

Initially, 18 fungicides were selected from literature and docked into the ubiquinone binding site by MOE v2013 (Molecular Operating Environment, 2017). The 2D-structures of the fungicides were constructed on Chemdraw (Li et al., 2004), converted into 3D and energy minimized by MOE. For docking, protein was minimized using AMBER10:EHT force field implemented in MOE (Gerber and Müller, 1995; Case et al., 2008). Similarly AMBER10:EHT was used to calculate charge and protein-ligand interactions (Molecular Operating Environment, 2017). All the heavy atoms were fixed until an RMSD gradient of 0.05 kcal mol^−1^ ^−1^ was achieved. Twenty conformations of each ligand were generated, of which top ranked (based on score) conformation was selected for analysis. The protein-ligand interactions were visualized by Chimera.

### Pharmacophore modeling and compound selection for virtual screening

Pharmacophore modeling was performed in order to screen the most appropriate from ZINC database. The pharmacophore model was generated by superimposing the 3D-structures of 18 fungicides, on the positions of annotation points like aromatic (ARO) center, H-bond donors (HBD) and acceptors (HBA), and hydrophobic centroids (HYD) using MOE. The derived pharmacophore model contained two HYD center, one HBD and one HBA (Supplementary Figure S5). The model was used to screen a set of 17,900,742 compounds, collected from ZINC (drug like category) database (Irwin et al., 2012). The pharmacophore based screening retrieved 50,000 compounds which were subsequently subjected to the SBVS protocol.

### Virtual screening by AutoDock tools

Initially 50,000 compounds were docked into the Qp site of SDH by ADT4. The docking calculations were performed on an Intel-Xeon-Quad™ core processor 3.0 GHz linux work station. Empirical free energy function and Lamarckian Genetic Algorithm was applied. The scoring function included the van der Waals interaction represented as a Lennard-Jones 6-12 dispersion/repulsion term, the hydrogen bonding represented as a directional 12-10 term, and the Coulombic electrostatic potential. Kollman charges were added on the protein model. Partial charges of ubiquinone were assigned with Gasteiger charges. The active site was defined on Qp site by AutoGrid with grid size and spacing of 70 × 70 × 70, and 0.375, respectively. Step sizes of 1.0 and 50° were set for translation and rotation, respectively. A number of energy evaluations were set to 250,000. Ten docked conformations of each compound were generated and the lowest energy conformation was selected for interaction analysis.

### Rescoring of compounds by MOE

Subsequently, top 1% compounds based on the ADT docking results were selected and re-scored by MOE. After which a re-scoring consensus strategy was adopted and the best compounds (based on ADT and MOE scores) were selected for visual inspection. The interactions analysis suggested that 25 compounds can be used as potential SDHIs. Out of 25 compounds, 12 were available; those were purchased for experimental testing.

### Experimental testing

#### Antifungal agents

Twelve antifungal agents from the ZINC database were used to prepare a stock solution at a concentration of 2.0 μgmL^−1^. Dimethylsulfoxide (DMSO) 1% was used as solvent in water. The final concentration of DMSO in all the assays was 0.1% (vol/vol). The solutions were stored at room temperature for antifungal activities.

#### Fungal strain

Virulent *A. solani* isolate accession number: NL03003 was kindly provided by Bert Evenhuis (Wageningen University and Research). Fungal strain was maintained on potato dextrose agar (PDA: potato extract = 4 g; dextrose = 20 g; agar = 15 g; (pH 6.6); H_2_O = 1,000 mL) plates at 27°C.

#### *In vitro* antifungal assay against *A. solani*

Antifungal activity of twelve selected compounds was evaluated against mycelial growth of the *A. solani* isolate NL03003 using agar well diffusion test (Magaldi et al., 2004; Valgas et al., 2007) on PDA. Four wells, each with 3 mm in diameter, were made in agar and filled with 50 μL of the antifungal agent. The same solution that dissolve the compounds was used as negative control, and penthiopyrad (a commercially available SDHI fungicide) was used as positive control. Fungal mycelium plugs (5 mm in diameter) of *A. solani* (NL03003) were removed from the actively growing margins of 7 days old cultures and placed at the center of the plate. The inoculated plates were incubated at 25°C for 5 days. Antimicrobial activity was estimated by measuring the zone of growth in comparison to the control. % Inhibition of fungal mycelial growth was calculated with respect to the control using the following formula:

$$\text{Percent Inhibition }\left(\%\right)=\frac{\text{C }-\text{ TX }100}{\text{C}}$$

Where C and T are average of three replicates of diameter of fungal colony (cm) in control and treatment plates, respectively. The experiment was conducted twice with three replicates for each treatment.

### *In vivo* antifungal potential

#### Inoculum preparation

The pathogen was cultured on V8 medium (V8 Juice = 200 mL, Water = 800 mL) by inoculating virulent *A. solani* isolate (NL03003) and incubating in shaking incubator at 28°C for 4–5 days until the culture became black. After 4–5 days, the culture was poured on PDA plates and exposed to black light (TL-D 18W BLB 1SL/25) for 12 h of light/dark. After 2–3 days, when the plates were completely dried, inoculum was prepared by flooding the plates with water and gently rubbing the surface. The final concentration of spore suspension was adjusted to 1 × 10^5^ spoers mL^−1^. The resulting suspensions was kept for 15–20 min to settle down the spores, water was poured off and equal volume of 1/5th strength PDA was added.

#### Pathogenicity test

Spore suspension (1 × 10^5^ spores mL^−1^) was used to inoculate 5 weeks old potato plant (cultivar Desirée) to confirm pathogenicity. The plants were homogenously sprayed with spore suspension and placed inside a tent in a climate cell (22–24°C, 16-h photoperiod). Immediately after inoculation, a fogger placed inside the tent was turned on for 24 h in order to create a humid environment (RH >99%). After the first 24 h, the fogger is only turned on during the nights and the humidity in the climate cell is kept around 70%. The pathogen–related symptoms were visually assessed after 5 days post-inoculation.

#### Antifungal potential of compounds on detached leaves

A detached leaf screening technique was used to evaluate the anti-fungal potential of 12 selected compounds. Fully expanded middle leaves were collected excising at the base of petiole from 5 weeks old potato plants. Leaves were placed in water-saturated florists foam (Oasis, Grunstadt, Germany) on top of moistened filter paper in a tray with abaxial surface up. Leaves were treated for: preventive, curative and eradicant activity. Compounds (1 μgmL^−1^) were applied with a sprayer until the leaf surface was thoroughly wet. The control leaves were similarly treated with 1% DMSO (negative control) and penthiopyrad (positive control). Each leaf was inoculated with two separate droplets (10 μL) of 1 × 10^5^ spores mL^−1^ spore suspension of *A. solani* (isolate: NL03003). The trays were then covered with transparent lids, transferred into a climate chamber, and incubated at 25°C for 5 days under 16 h photoperiod at >95% relative humidity (RH). Assessment of EB disease symptoms was conducted 5 days after inoculation by measuring the lesion diameter. The % protection was calculated by:

$$\text{Control Efficacy }\left(\%\right)=\frac{\text{C }-\text{ TX }100}{\text{C}}$$

Where C and T are the average of four replicates of lesion diameter (mm) in control and treatment plates, respectively. The experiment was conducted twice with four replicates for each treatment.

#### Preventative activity

One day before inoculation of spore suspension of *A. solani*, a preventative spray was carried out with 12 compounds. Five days after inoculation, the % disease control was assessed.

#### Curative activity

A curative spray was carried out with twelve selected compounds on the same day of inoculation of *A. solani* spore suspension. Five days after inoculation, the % disease control was assessed.

#### Eradicant activity

The compounds were sprayed 1 day after inoculation of *A. solani* spore suspension and the % disease control was assessed after 5 days of inoculation (= 4 days after spraying).

#### Fungi-toxic effect of compounds against early blight (greenhouse studies)

Murashige and Skoog (MS20) 1962 basal medium supplemented with vitamins, sucrose (20 gL^−1^) and micro agar (15 gL^−1^) was used to propagate potato plants. The plant shoots were cut into internodes of about 1–2 cm across each section containing a node. The explants were transferred to MS medium. All the cultures were maintained at 25°C under 16 h light/8 h dark for 2 weeks. Once roots were well developed, the plants were transferred into pots (9 × 9 × 10 cm) containing a clean light potting soil. Plants were watered depending on the weather and green-house conditions for 5 weeks under long day conditions. The *in vivo* fungi-toxic effect of the compounds against *A. solani* isolate (NL03003) was conducted as; preventive, curative and eradicant activity.

The control plants were treated with 1% DMSO (negative control) and penthiopyrad (positive control). Compounds (1 μgmL^−1^) were applied with a sprayer until the leaf surface was thoroughly wet. Four leaves in the middle third of the plant canopy were inoculated on each plant and each leaf was inoculated with two droplets (10 μL) of 1 × 10^5^ spores mL^−1^ of *A. solani* (NL03003). The treated plants were held in mist chambers (>95% relative humidity, 22–24°C, 16-h photoperiod). EB disease assessment was conducted 5 days after inoculation by measuring the diameter of lesion. Mean lesion diameter from four leaves each having two inoculation points on single plant was considered as one replicate. A randomized complete block design with 4 replications was used for the experiment. Experiment was repeated twice. The %disease control was calculated by:

$$\text{Disease control }\left(\%\right)=\frac{\text{C }-\text{ TX }100}{\text{C}}$$

Where C and T are average of four replicates of lesion diameter (mm) in control treatment plates, respectively.

#### Antifungal potential of compounds on potato tubers

The anti-fungal potential of 12 SDHI's was tested on potato tubers using the modified method reported by Scuderi et al. (2009). Tubers were purchased from Ecoflora (www.ecoflora.be). Tubers were disinfected by immersion in 3% sodium hypochlorite for 2 min, rinsed three times in sterile deionized water, and air dried for 2 h. Artificial inoculation was carried out by removing a disc of 3 mm × 3 mm from the tuber using a cork borer and replacing it with an agar plug of same size from a 5 days old culture of the fungal pathogen. The tubers were sprayed with the compound as preventive, curative and eradicant antifungal activity. Compounds were applied with a sprayer until discs were thoroughly wet. Control tubers were sprayed with 1% DMSO (negative control) and penthiopyrad (positive control). Then, tubers were placed on a mesh platform in plastic trays. Water (500 mL) was added in each tray and covered to maintain high relative humidity and incubated at 24°C for 21 days. Four tuber replicates, each with three inoculation points, were used. Disease assessment was conducted at 21 days post inoculation by measuring the diameter of the lesion. The mean diameter from three lesions on a single tuber was considered as one replicate. The experiment was repeated twice.

#### Succinate dehydrogenase activity (colorimetric assay)

SDH activity colorimetric assay was performed with SDH colorimetric assay kit 1/14 (Catalog # K660-100, purchased from BioVision). Fungal mycelium (10 mg) was rapidly homogenized with 100 μL ice cold SDH assay buffer, kept on ice for 10 min and centrifuged at 10,000x g for 5 min. To the supernatant, 15 μL of sample and 5 μL (10 μgmL^−1^) of each compound per well was added and the volume was adjusted to 50 μl with SDH assay buffer. For the SDH positive control, 15 μL of SDH positive control was pipetted into the desired well(s) and the final volume was adjusted to 50 μL with SDH assay buffer. 0, 4, 8, 12, 16, and 20 μl of 2 mM DCIP Standard was added into a series of wells in 96-well plate to generate 0, 8, 16, 24, 32, and 40 nmol/well of DCIP Standard. The volume was adjusted to 100 μL/well with SDH assay buffer. A reaction mixture of 50 μl was prepared containing 46 μl SDH assay buffer, 2 μl SDH substrate mix, and 2 μl SDH Probe. Reaction mix of 50 μl was added to each well containing the samples and positive control and mixed well. The absorbance was measured spectrophotometrically immediately at 599 nm in kinetic mode for 2 h at 25°C. The SDH activity of the test sample was calculated as ΔOD = A1–A2. The ΔOD was applied to the DCIP standard curve to get B nmol of DCIP reduced during the reaction time (ΔT = T2–T1). SDHI activity was measured using the formula:

$$\text{Sample SDH Activity=}\frac{\text{B}}{(\Delta \text{T X V})\;\times \text{ Dilution Factor}}$$

Where: B = amount of reduced DCIP from Standard Curve (nmol), ΔT = reaction time (min.), V = sample volume added into the reaction well (μl), D = Dilution Factor.

#### Statistical analysis

The results of the detached leaf assay and green house assay were analyzed in a 2-factorial ANOVA. Additionally, 12 SDHI's were analyzed using Tukey's HSD, to indicate significant differences between groups. All statistical analyses were performed with the statistical package Statistix 8.1(Analytical Software, 2005).

## Results

### Homology modeling of *A. solani* SDH

Initially homology modeling of *A. solani* SDH was conducted. SDH is composed of two hydrophilic proteins: flavoprotein (Fp) and iron- sulfur protein (Ip), and two trans-membrane proteins, i.e., large cytochrome b (CybL) and small cytochrome b (CybS), and prosthetic groups required for electron transfer from succinate to ubiquinone. The soluble catalytic heterodimer is composed of subunit A (SDHA) and B (SDHB). SDHA contains coenzyme FAD, SDHB contains three iron-sulfur clusters: [2Fe-2S], [4Fe-4S], and [3Fe-4S] (Hägerhäll, 1997). The membrane spanning region contains one or two hydrophobic peptides with or without heme groups. SDH is classified into five types (A–E) according to the number of their hydrophobic subunits and heme groups (Lemos et al., 2002). The mitochondrial SDH belong to type C that contains one heme molecule and two trans-membrane proteins: CybL or subunit C (SDHC) and CybS or subunit D (SDHD) (Lemos et al., 2002). To date, only the structures of prokaryotic SDH (one for SDHB, one for SDHD, and one for SDHC), which share a similar enzymatic function with mitochondrial SDH (Complex II), have been reported (Iverson et al., 1999; Lancaster et al., 1999; Yankovskaya et al., 2003). The 3D model of *A. solani* SDH was constructed *via* homology modeling. The structural features were characterized and stereo-chemical properties were evaluated. The quality of the model was scrutinized by PROCHECK (Laskowski et al., 1993), ERRAT plot and verify 3D. The Ramachandaran plot showed that 92% of the residues are located in the most favored regions while only 0.2% residues were found in disallowed region however they are not involved in ligand binding (Supplementary Figure S3). The topology and packing of six helices of model were similar to templates. The model showed 82% quality factor *via* ERRAT plot (Supplementary Figure S4) and the Verify 3D score was 0.2. The overall results showed that the model is of good quality and can be used in the VS studies.

### Structural overview

**Figure 1:**
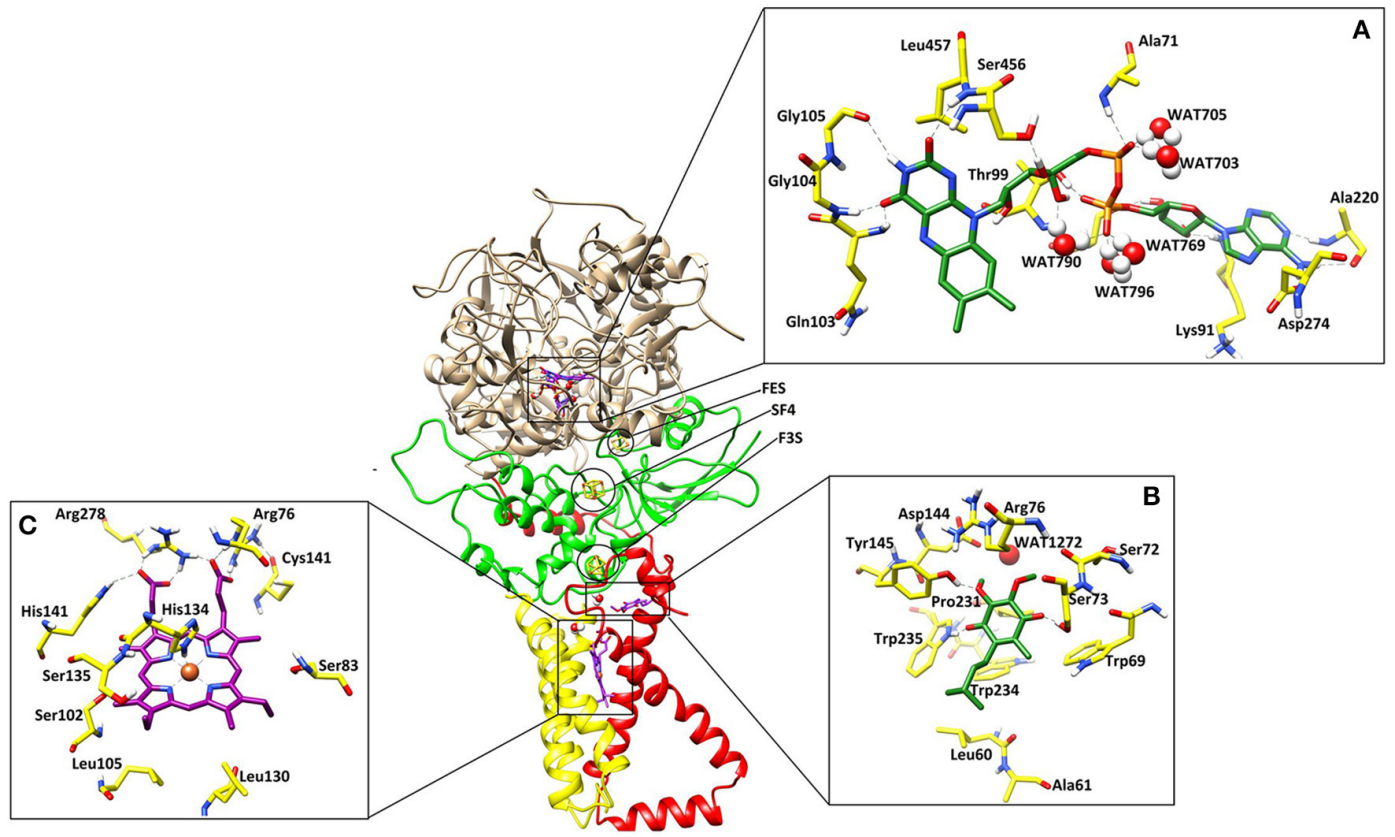
The three dimensional structure of the *A. solani* SDH model. SDHA (Fp), SDHB (Ip), SDHC (CybL) SDHD (CybS) are depicted in tan color, green, red, and yellow colors, respectively. **(A)** Binding interactions of FAD. The ligand and the surrounding residues are depicted in Green and Yellow sticks, respectively **(B)** Binding interactions of Ubiquinone. The interacting residues are shown in Green and Yellow sticks, respectively **(C)** Binding interactions of Heme. The interacting residues are shown in Purple and Yellow sticks, respectively. The Hydrogen bonding are displayed in Green dotted lines.

The 3D-structure of model is comprised of four proteins chains: FAD binding protein/flavoprotein (SDHA or Fp, 600 residues), iron-sulfur protein (SDHB or Ip, 235 residues), and two membrane-anchor proteins (SDHC or CybL, 94 and SDHD or CybS, 101 residues) with a total of six trans-membrane helices (Figure 1). The overall 3D- structure of the protein is “q” letter shaped with a hydrophilic head and a hydrophobic multi-pass trans-membrane-anchor tail. The hydrophilic head is composed of SDHA, and SDHB. The interactions between four SDH subunits are essential for its formation, stability, and function. No direct interaction between SDHA and the membrane spanning region was observed, however the contact surfaces of the four subunits are dominated by the hydrophobic interactions.

SDH is a hetero-tetramer complex (Lancaster, 2002). Functionally SDH possess three domains: SDHA and B (located in mitochondrial matrix) play role as catalytic domain and, electron transfer subunit, respectively. While the third domain composed of SDHC and D, form dimeric membrane spanning region, involved in heme binding. SDHA and SDHB show hydrophilic characteristic where they are attached to the inner cytoplasmic surface of the membrane. Both SDHA and SDHB interact with SDHC and SDHD hydrophobically. SDHA and SDHB are more structurally conserved with higher sequence similarity while SDHC and SDHD have higher sequence variation among different organisms (Tran et al., 2006). In order to be anchored in the membrane, this domain must have hydrophobic residues (White and Wimley, 1999; Arce et al., 2009).

### Prosthetic groups

**Figure 2:**
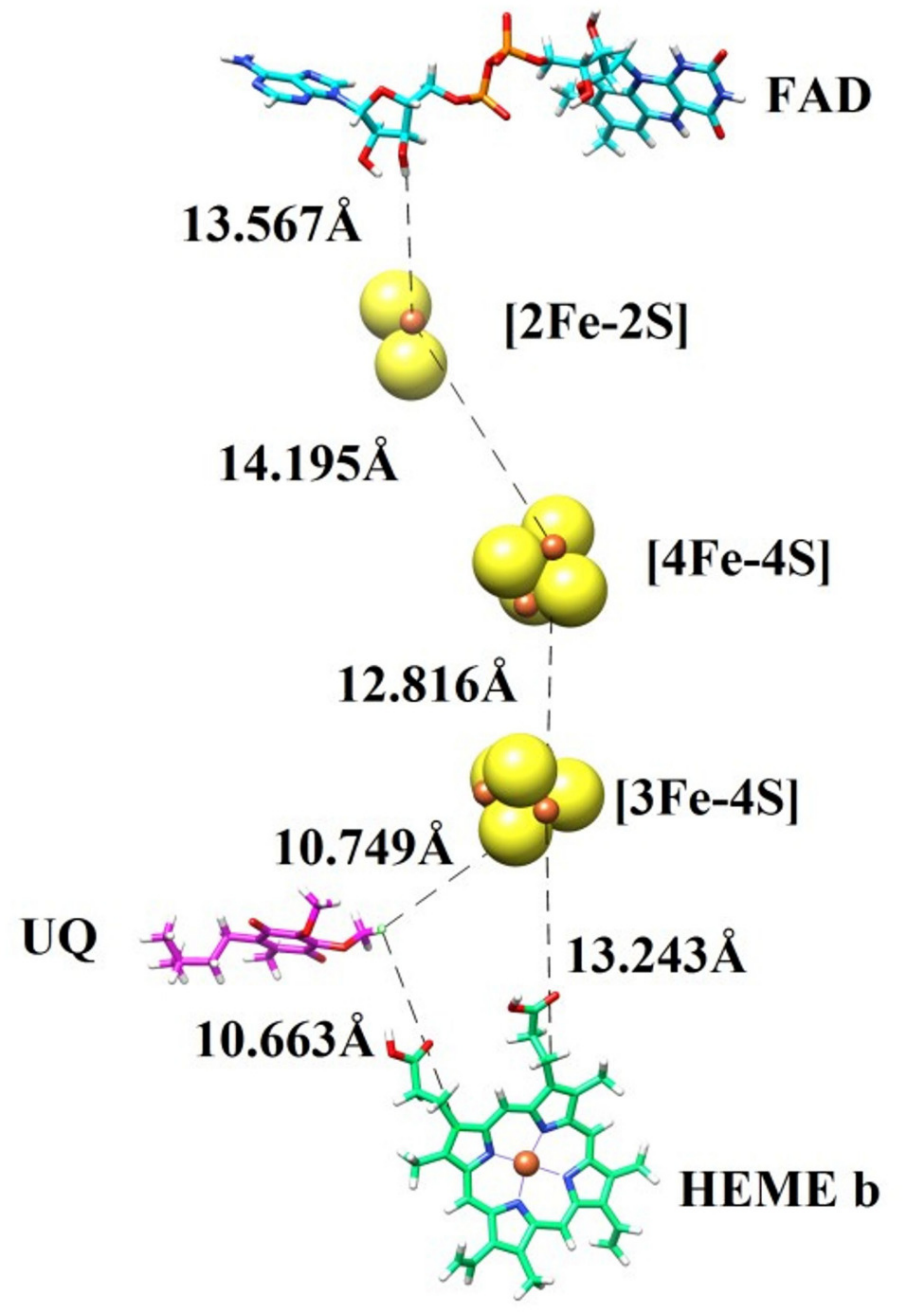
The prosthetic groups constituting the electron transfer pathway (FAD, [2Fe-2S], [4Fe-4S], [3Fe-4S], and heme b) are shown together with ubiquinone (UQ), along with their edge-to-edge distances.

In order to transfer electron from succinate to ubiquinone, five prosthetic groups (FAD, [2Fe-2S], [4Fe-4S], [3Fe-4S], and heme) are required in SDH structure. These prosthetic groups along with ubiquinone are arranged in a linear path with favorable distances to transfer electron. The binding modes of prosthetic groups in our model are in accordance with those found in templates. The model and templates share near-equivalent positions for FAD, and the three iron-sulfur clusters in the hydrophilic head, and one heme moeity in the hydrophobic tail. The edge-to-edge distances between FAD and the 2Fe-2S cluster, and between the different iron-sulfur clusters, are each less than 14.2, which is favorable for direct electron transfer (Page et al., 1999). Classical dogma asserts that electrons are transferred from succinate to ubiquinone through FAD, [2Fe-2S], [4Fe-4S], [3Fe-4S], and heme b sequentially. However, the observed distances between these redox centers in SDH suggests that it would not be favorable for heme b to transfer electron from [3Fe-4S] to ubiquinone, as observed in the template structure. The edge-to-edge distance is about 10.749 between the bound ubiquinone and [3Fe-4S], about 13.243 between [3Fe-4S] and heme b, and about 10.663 between heme b and ubiquinone. These distances suggest that it is not necessary for electrons to transfer to ubiquinone through a mediator heme b (Figure 2).

In general, SDHA catalyzes oxidation of succinate to fumarate. SDHA contains a FAD cofactor, which is reduced to FADH_2_ by losing two electrons in a process. Electrons from SDHA are transferred to SDHB *via* the iron sulfur cluster. These electrons are then transferred to ubiquinone which is bound to SDHC and SDHD, reducing it to ubiquinol. A heme molecule is placed between SDHC and SDHD (Oyedotun and Lemire, 2004).

### FAD binding site

The cofactor FAD was manually docked into model by superimposing the catalytic dimer of 1ZOY on the model since cofactor locations are highly conserved among the catalytic dimers. FAD is located at the interface of the β barrel subdomain and embedded subdomain of SDHA and is directed into the β barrel. The interacting residues are highly conserved. The flavin moiety is engaged in H-bonding with Gln103 (1.82), Gly104 (1.50), Gly105 (2.52), Ser456 (1.59), Leu457 (2.18), and WAT790 (1.56). The α-PO_4_ mediates interaction with Ala71 (2.21), WAT703 (1.27), and WAT705 (1.23), while β-PO_4_ interacts with Thr99 (1.32), WAT769 (1.31), WAT790 (1.56), and WAT796 (1.33). The ribose sugar forms H-bond with Ser97 (1.61) and Lys91 (1.67). The adenine nucleotide interacts with the Ala220 (1.81) and Asp274 (1.39). Hence five water molecules stabilize the cofactor in the binding site. The 3D-interactions are depicted in Figure 1A.

### The iron-sulfur clusters

**Figure 3:**
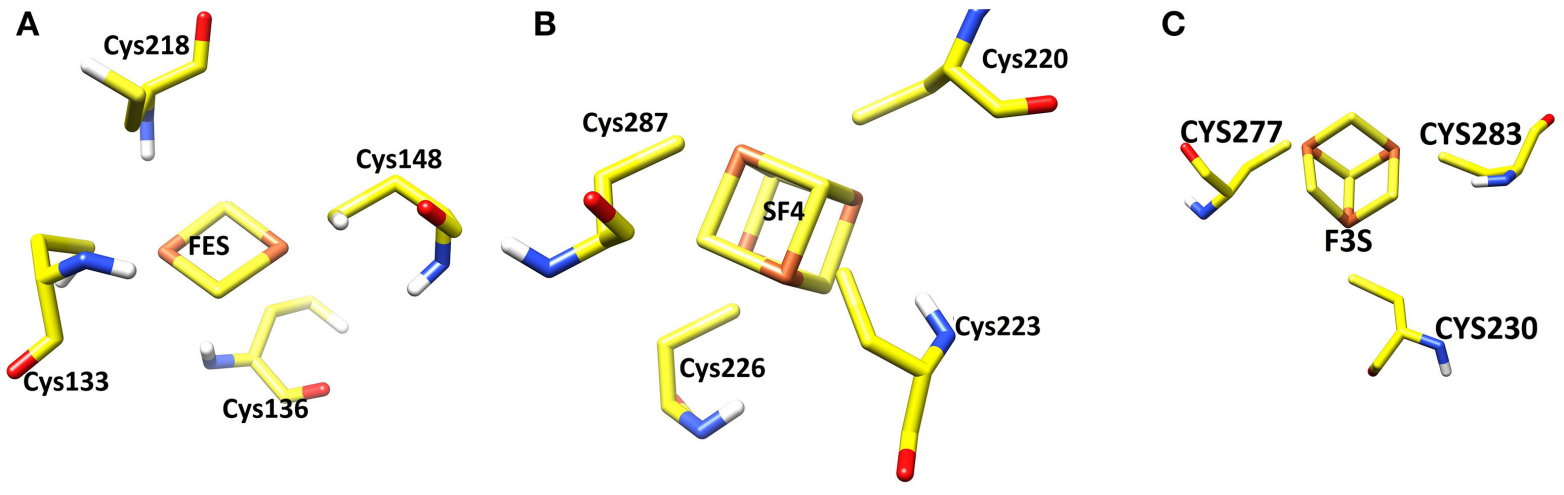
Three iron sulfur clusters present in the model. **(A)** FES **(B)** SF4 **(C)** F3S.

Three iron-sulfur clusters are well coordinated by cysteine residues. F3S is ligated with three conserved cysteine: Cys-230, Cys-277, and Cys-283. FES binds at the loop region by four cysteines: Cys-128, Cys-133, Cys-136, and Cys-148. SF4 is ligated with Cys-220, Cys-223, Cys-226, and Cys-287. All these cysteines are highly conserved in the structure (Figure 3).

### Quinone binding site

Quinone binding site is a hydrophobic pocket comprised of residues from SDHB, SDHC, and SDHD (Horsefield et al., 2006) located within the membrane spanning region. To date, all the identified SDH structures contain at least one heme group and quinone reduction site (Oyedotun and Lemire, 2004). Quinone forms a direct H-bond with Trp235 of SDHB (1.73) and Tyr145 of SDHD (1.77) while one H-bond with Ser73 of SDHC (1.60) (Figure 1B). The quinone is inserted in the model into the cleft formed by Arg76, Tyr145, and Trp234. The hydrophobic interactions are provided by Tyr145, Pro231, Trp234, and Trp235. The environment around this region is similar in both eukaryotic and prokaryotic SDH structures, and substrate binding and catalytic domains are well conserved. H-bonds between ubiquinone and residues Ser73, Tyr145, and Trp234 contribute to the binding specificity, and are suggested to play functional roles in the protonation of ubiquinone upon reduction (Yankovskaya et al., 2003).

### Heme binding site

Heme b was placed into the model SDH by superimposing on templates. In this model, His134 forms coordinate bond with heme iron at a distance of 1.88, which is within the range of coordination distances observed in the *E. coli* SDH and *W. succinogenes* FRD structures. Arg76 mediates two H-bond with heme (1.24 and 1.46) while several H-bonds are mediated by Arg278 (1.33, 1.41, and 1.42). His141 also forms H-bond at a distance of 2.56 (Figure 1C). Additionally Ser83, Ser102, Ser135, Leu105, Leu130 provides hydrophobic interactions to the molecule.

### Binding modes of 18 known fungicides

To further confirm the role of binding residues, eighteen known inhibitors were docked into the model (Supplementary Figure S6). The structures and docking scores of known SDHIs are tabulated in Supplementary Table S2. Benodanil forms H-bond with Ser73 (2.22). The binding energy was −9.45. Bnzovindiflupyr (−9.69) formed two H-bonds with Arg76 (3.07 and 3.04) and one with Trp235 (1.98). Bixafen (−9.88) formed multiple H-bonds with Arg76 and one with Ser73. Boscalid (−10.48) is H-bonded with WAT1272 (2.48) and formed two H-bonds with Arg76 (2.34 and 1.63). Carboxin (−10.25) formed two H-bonds with Arg76 (2.0 and 2.06) *via* oxathine moiety, and one with Trp235 (2.98). Fenfuram (−9.03) formed two H-bonds with Arg76 (2.74 and 2.17). Fluopyram (−10.03) mediates H-bonding with Arg76 (2.77) and Trp235 (1.74). Flutolanil (−9.74) mediates hydrophobic interactions with Leu60, Trp69, Tyr145, and Trp234. Fluxapyroxad (−9.96) formed H-bond with Trp69 (2.43), Ser73 (2.240), Arg76 (2.43), and Trp235 (2.88). Furametpyr's benzofuram moiety binds with Ser73 and Tyr145 *via* H-bonds with bond length 2.98 and 3.05, respectively. Isofetamid (−10.46) is H-bonded with Ser73 (2.03), Arg76 (2.79), and Tyr145 (2.63). Isopyrazam (−9.58) mediates H-bond with Arg76 (2.12), moreover pyrazole nitrogen forms two H-bond with Arg76 (2.33 and 2.60), while amide oxygen of isopyrazam is H-bonded with Ser73 (2.17). Mepronil (−10.51) is H-bonded to Ser73 (2.30) and Trp235 (2.67). Oxycarboxin (−11.07) formed H-bond with Arg76 (1.42). Penflufen (−9.56) mediates three H-bonds with the Arg76 (2.29, 0.96, and 2.47) and one with Trp235 (1.30). Penthiopyrad (−9.47) mediates one H-bond with Trp235 (2.29), Tyr145 (2.94), and Ser73 (2.69) while two H-bonds with Arg76 (2.64 and 2.79). Sedaxane (−10.13) forms multiple H-bonds including three with Arg76 (2.99, 2.32, and 2.72), two with Trp235 (1.49 and 2.92) and one with WAT1272 (2.65). Thifluzamide (−11.68) formed H-bond with Arg76 (2.36), Ser73 (1.98), Ser76 (1.94) and two with WAT1272 (2.76 and 3.01). The interaction analysis revealed that Ser73 and Arg76 are crucial residues for ligand binding, while Leu60, Trp69, Tyr145, Pro231, Trp234, and Trp235 provides hydrophobic interactions to the ligands. Moreover some water molecules are also involved in protein-ligand bridging.

### Post-screening analysis and SDHIs selection for experimental testing

50,000 compounds were obtained post-pharmacophore based screening, docked at quinone binding site of model. At first step, docking was conducted by ADT. The time to dock one ligand was ~1–2 min for ADT. Subsequently top 1% compounds (based on ADT results) were selected and re-scored by MOE suit (Molecular Operating Environment, 2017). After re-scoring consensus strategy was adopted and the best suggested compounds (based on ADT and MOE scores) were selected to analyze their interaction. The interactions analysis suggested 25 compounds as potential hits. Out of 25 compounds, 12 were commercially available; those were purchased and evaluated by *in vitro* and *in vivo* testing. The docking and rescoring results are tabulated in Supplementary Table S3.

### Interactions of selected SDHIs with SDH model

**Figure 4:**
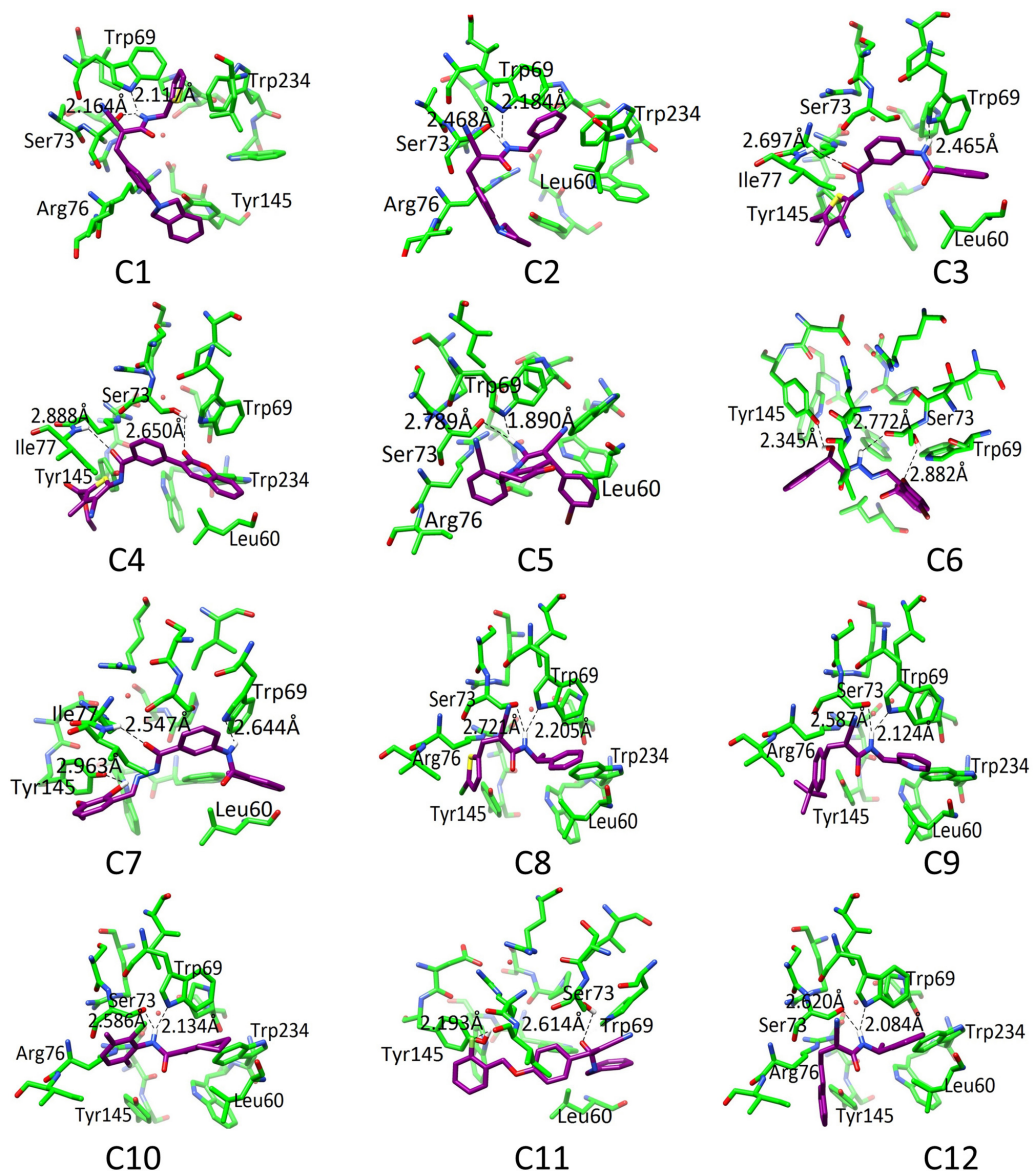
The docked orientation of 12 SDHIs. The interacting residues and the compounds are presented in green and magenta colors, respectively. H-bonds are displayed in black dotted lines.

The binding pattern revealed that all twelve inhibitors bind at the ubiquinone binding site (Figure 4). The compound **C1** formed H-bond with Ser73 (2.61) and Trp69 (2.12). The **C2** formed H-bonds with Ser73 and Trp69 with bond length 2.47 and 2.18, respectively. The **C3** revealed H-bond with Trp69 (2.46) and Ile77 (2.69). The **C4** mediated H-bond with Ser73 (2.65) and Ile77 (2.89). The **C5** formed a H-bond with Ser73 (2.79) and Trp69 (1.89). The **C6** mediates H-bond with Tyr145 (2.34), Ser73 (2.77 and 2.88). The **C7** formed H-bond with Tyr145 (2.96), Ile77 (2.46), and Trp69 (2.64). The **C8** formed H-bonds with Trp69 and Ser73 at a distance of 2.21 and 2.72, respectively. The **C9** is H-bonded with Trp69 and Ser73 at the distance of 2.12 and 2.59, respectively. The **C10** mediates H-bonds with Trp69 (2.13) and Ser73 (2.59). The **C11** is H-bonded with Ser73 (2.61) and Tyr145 (2.19). The **C12** formed H-bonds with Trp69 (2.08) and Ser73 (2.62). These observations showed that all the hits binds with Ser73 *via* H-bond or hydrophobic interaction; confirms the crucial role of Trp69, and Ser73 in ligand binding and stabilization in the active site. However, Leu60, Trp69, Ser73, Arg76, Ile77, Tyr145, and Trp234 provide hydrophobic interactions to the compounds.

### Diffusion assay

The antifungal potential of 12 hits was evaluated *in vitro* by a diffusion assay. All compounds were effective in reducing the linear mycelial growth of *A. solani* at 2 μgmL^−1^ (Table 1). Antifungal potential was evaluated by an agar diffusion test after the incubation of 7 days. The results showed that compounds **C1**, **C6**, and **C10** were most effective, exhibiting ≥80% inhibition in the growth of fungi and also significantly suppressing fungal biomass as compared to commercially available SDHI, penthiopyrad. Compound **C2** and **C9** exhibiting 79.95% and 78.34% inhibition while **C3** and **C12** were found moderately effective with ≥71% inhibition. The results depict that **C10** is the most potent compound amongst twelve tested SDHIs. The compounds **C4**, **C8** and **C11** showed ≥69% reduction in fungal mycelia growth. The compounds **C5** and **C7** were relatively less effective in controlling *A. solani* with % inhibitions of ≥65%, however the least antifungal activity was exhibited by **C5** (≥62%), while still higher than penthiopyrad (positive control). These results proved that the selected hits possess ≥60% suppressive effect against mycelial growth of *A. solani*.

**Table 1:** *In Vitro* antifungal potential of SDHIs against *A. solani* as assessed through agar diffusion assay.

**Treatments (2 μgmL^−1^)**	**Colony diameter (cm)**	**Percentage inhibition (%)**
DMSO	8.644 a (± 0.029)	0
Penthiopyrad	3.221 b (± 0.082)	62.737
C1	1.693 d (± 0.058)	80.414
C2	1.733 d (± 0.047)	79.95
C3	2.449 c (± 0.058)	71.668
C4	2.628 c (± 0.049)	69.59
C5	2.977 b (± 0.044)	65.55
C6	1.721 d (± 0.051)	80.09
C7	2.966 b (± 0.05)	65.68
C8	2.663 c (± 0.032)	69.192
C9	1.872 d (± 0.038)	78.343
C10	1.671 d (± 0.049)	80.668
C11	2.599 c (± 0.074)	69.932
C12	2.471 c (± 0.048)	71.413

*Values in parentheses indicate standard error. Means sharing the same letters are not significantly different (P < 0.05; LSD, Statistix 8.1)*.

### Pathogenicity test

The pathogenicity test revealed that the initiation of typical symptoms of the disease appeared at 5 days post-inoculation on potato leaves. Inoculated plants showed the symptoms of EB. Control plants remained healthy and showed no symptoms.

### Detached leaf assay: preventive, curative, and eradicant treatment

The antifungal activity of 12 compounds was investigated against *A. solani* on detached leaves of potato plants. During the preventive treatment, all the compounds reduced *in vivo* growth of *A. solani* on potato leaves and the pre-treated detached leaves all showed a reduced lesion diameter (Table 2). The compounds **C1**, **C2**, **C6**, and **C10** were most effective and exhibited ≥80% disease control, while **C7**, **C11**, and **C12** exhibited moderate lesion expansion. The compounds **C3**, **C4**, **C5**, and **C8** showed reduction in lesion diameter in a range of 70–69% while **C9** was appeared as least effective with ≥68% disease control.

**Table 2:** Comparison of disease control efficacy of SDHIs on detached leaves of Potato.

**Compounds**	**Treatment**		
	**Pre-treatment**	**Co-treatment**	**Post-treatment**
	**Disease control (%)**	**Disease control (%)**	**Disease control (%)**
DMSO	0 a (± 0.108)	0 a (± 0.15)	0 a (± 0.093)
Penthiopyrad	68.086 b (± 0.135)	63.021 b (± 0.131)	60.402 b (± 0.158)
C1	80.508 c (± 0.097)	75.739 ef (± 0.136)	68.765 cd (± 0.13)
C2	80.207 c (± 0.065)	71.005 de (± 0.156)	68.572 cd (± 0.155)
C3	70.564 b (± 0.139)	69.68 cd (± 0.133)	64.17 bc (± 0.15)
C4	69.796 b (± 0.124)	65.483 bc (± 0.158)	60.541 b (± 0.164)
C5	70.96 b (± 0.118)	68.585 bc (± 0.195)	61.702 b (± 0.18)
C6	80.405 c (± 0.062)	75.495 ef (± 0.177)	70.266 d (± 0.183)
C7	71.087 b (± 0.098)	69.183 cd (± 0.163)	63.497 bc (± 0.16)
C8	69.951 b (± 0.1)	64.402 bc (± 0.166)	63.35 bc (± 0.201)
C9	68.68 b (± 0.136)	65.483 bc (± 0.119)	61.222 bc (± 0.135)
C10	81.497 c (± 0.066)	77.372 f (± 0.105)	73.454 d (± 0.101)
C11	71.601 b (± 0.11)	65.143 bc (± 0.123)	62.189 b (± 0.19)
C12	71.799 b (± 0.145)	64.055 bc (± 0.12)	59.767 b (± 0.148)

*Values in parentheses indicate standard error. Means sharing the same letters are not significantly different (P < 0.05; LSD, Statistix 8.1)*.

During curative treatment **C10** was most effective which showed ≥73% control of EB. However, **C1**, **C2**, and **C6** also significantly suppressed lesion development with ≥70% inhibition while **C8** and **C9** were less effective in control of *A. solani* in co-treatment of detached leaves of potato.

The detached leaves of potato plants were sprayed with compounds after 24 h of artificial inoculation with spore's suspension of *A. solani*. The compounds **C1**, **C2**, **C6**, and **C10** exhibited 68–73% reduction in lesion diameter caused by *A. solani*. The compounds **C3**, **C7**, **C8**, **C11**, and **C12** exhibited moderate antifungal activity during eradicant treatment. While compound **C4** was least effective and was not significantly different from penthiopyrad. The results are depicted in Table 2. **C1**, **C2**, **C6**, and **C10** decreased lesion development in all the three treatments, while rest of the compounds showed 59–50% reduction in disease development. Tukey's HSD all pairwise comparison showed three homogeneous groups of preventive, curative and eradicant treatment and all three means were significantly different from one another.

### Efficacy of SDHIs on EB under greenhouse conditions: preventive, curative and eradicant treatment

**Figure 5:**
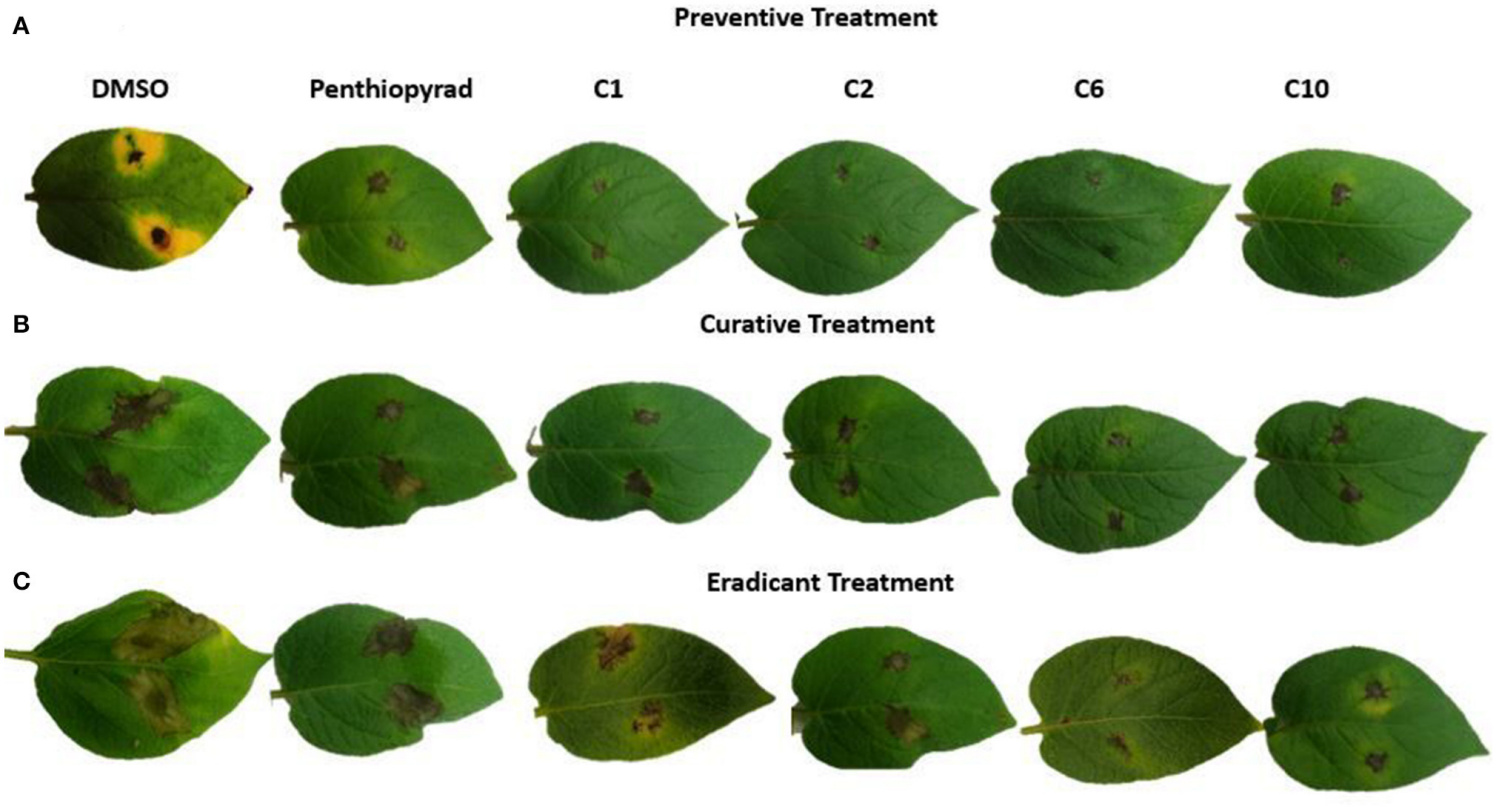
Symptoms of *A. solani* infection on spot-inoculated potato leaves treated with **C1**, **C2**, **C6**, and **C10** at 5 dpi **(A)** Preventive treatment **(B)** Curative treatment **(C)** Eradicant treatment.

Twelve SDHIs were further evaluated in the greenhouse to control *A. solani* on whole potato plants. After preventive treatment, **C1**, **C2**, **C6**, and **C10** decreased lesion formation by *A. solani* with ≥80% (Table 3 and Figure 5A). The data clearly showed that significant disease control was achieved in pre-treated plants as compared to the positive control. Control efficacy was observed in a range of 65-83% when compounds were sprayed 24 h before inoculation (Table 3). All the compounds showed higher control efficacy as compared to penthiopyrad. However, **C9** showed minimum suppression in lesion development (≥65%).

**Table 3:** Comparison of lesion diameter and % disease (EB) control after treatment with SDHIs on potato leafs under Greenhouse conditions.

**Compounds**	**Treatment**					
	**Pre-treatment**		**Co-treatment**		**Post-treatment**	
	**Lesion diameter (mm)**	**Disease control (%)**	**Lesion diameter (mm)**	**Disease control (%)**	**Lesion diameter (mm)**	**Disease control (%)**
DMSO	12.933 a (± 0.132)		13.132 a (± 0.132)		12.960 a (± 0.099)	
Penthiopyrad	4.167 b (± 0.083)	67.803	4.848 bc (± 0.119)	63.082	5.783 b (± 0.111)	55.37
C1	2.543 c (± 0.111)	80.337	3.381 de (± 0.076)	74.253	3.895 d (± 0.16)	69.945
C2	2.383 c (± 0.148)	81.574	3.542 d (± 0.0716)	73.027	3.841 d (± 0.115)	70.362
C3	3.907 b (± 0.137)	69.79	4.221 c (± 0.1845)	67.857	5.176 bc (± 0.147)	60.061
C4	4.002 b (± 0.154)	69.055	4.63 bc (± 0.156)	64.742	5.627 bc (± 0.095)	56.581
C5	3.924 b (± 0.123)	69.659	4.909 b (± 0.182)	62.618	5.2433 bc (± 0.221)	59.544
C6	2.210 c (± 0.177)	82.911	2.818 e (± 0.089)	78.540	3.585 d (± 0.098)	72.337
C7	3.718 b (± 0.172)	71.125	4.356 bc (± 0.124)	66.829	5.437 bc (± 0.126)	58.047
C8	3.976 b (± 0.131)	69.256	4.698 bc (± 0.154)	64.224	5.295 bc (± 0.145)	59.143
C9	4.172 b (± 0.178)	67.414	4.549 bc (± 0.144)	65.359	5.026 c (± 0.111)	61.219
C10	2.579 c (± 0.102)	80.058	3.059 de (± 0.074)	76.705	3.776 d (± 0.135)	70.864
C11	3.685 b (± 0.134)	71.506	4.701 bc (± 0.099)	64.201	5.246 bc (± 0.121)	59.521
C12	3.878 b (± 0.136)	70.014	4.971 b (± 0.159)	62.145	5.495 bc (± 0.131)	57.6

*Values in parentheses indicate standard error. Means sharing the same letters are not significantly different (P < 0.05; LSD, Statistix 8.1)*.

Upon curative treatment, a lower control efficacy was observed on plants that received applications of compounds at the time of inoculation ranging from 62 to ≥78%. **C6** was most potent in the EB control with ≥78% disease control, while **C1**, **C2**, and **C10** also showed promising results with ≥ 70% disease control. **C12** was least effective in reducing lesion development (≥62%).

During eradicant application (spray of compounds 1 day after inoculation), **C6** appeared effective (Table 3, Figure 5B). Table 3 indicate that potato plants that received **C1**, **C2**, **C6**, and **C10** applications showed significant reduction in lesion diameter as compared to control, while **C3**, **C5**, **C8**, **C9**, and **C12** also were found to be effective against *A. solani*. However, **C4** showed the least promising results (≥57% reduction).

Application of **C1**, **C2**, **C6**, and **C10** at 1 μgmL^−1^ 24 h before inoculation of plants showed good control efficacy (Table 3, Figure 5C). All the new SDHIs (**C1**–**C12**) significantly reduced EB as compared to the control. Similarly, protective efficacy of the compounds was higher as compared to the curative and eradicant efficacy. However, the compounds depicted less eradicant efficacy.

Preventive treatment of all the compounds significantly decreased total lesion diameter by ranging from 2.21 to 4.172 mm compared to the control. Plants treated with compound before inoculation of *A. solani* showed consistently lower lesion development as compared to the other treatments and negative control. The results demonstrate that EB was adequately controlled by preventive application of compounds **C1**, **C2**, **C6**, and **C10**. Similarly these compounds were the most efficient in disease control in the plants sprayed with and after inoculation, however with lower efficiency in curative and eradicant treatment. The compound **C9** showed lower efficiency, however statistically equal to the positive control (67.414 and 67.903% respectively).

Higher reduction in lesion diameter was obtained by pre-treating the plants with **C6** at 1 μgmL^−1^. The compounds **C2**, **C6**, and **C10** showed the best disease control in plants after inoculation while all the compounds significantly controlled EB infection on potato as compared to the negative control. The results indicate that, during preventive measure, the compounds showed maximum protection against *A. solani*. The new SDHIs, particularly **C1**, **C2**, **C6**, and **C10**, can be effectively used to manage EB of potato.

### Main factor effects

The results of the greenhouse experiments, the analysis of variance for main factor effects and interactions showed that preventive spray treatment and compounds were significant determinants of control efficacy. There were significant differences among spray treatment and among compound treatments for control efficacy and lesion development (Table 3).

### Phyto-toxicity analysis

The tested SDHIs did not show any phyto-toxicity symptoms such as chlorosis, necrosis, wilting, scorching, hyponasty and epinasty on 1, 3, 5, and 7 days after application.

### Tuber assay: preventive, curative, and eradicant treatment

**Figure 6:**
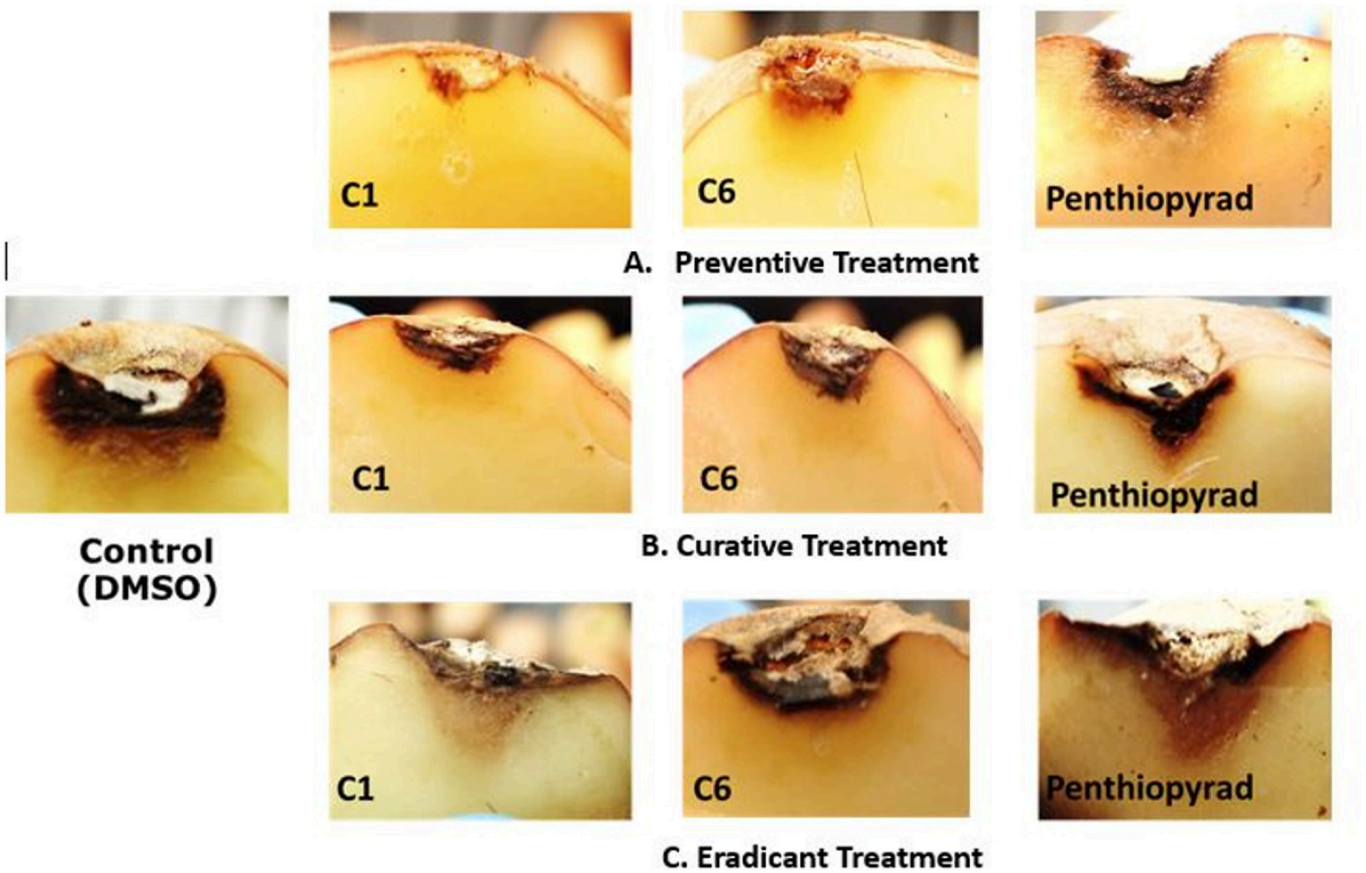
Symptoms of *A. solani* infection on inoculated potato tuber treated with **C1** and **C6** at 21 dpi **(A)** Preventive treatment **(B)** Curative treatment **(C)** Eradicant treatment.

The efect of SDHIs on EB development in potato tubers is shown in Table 4 and Figure 6. The **C1**, **C2**, **C6**, **C9**, and **C10** were most efficient in control of EB progression on potato tubers, with ≥75% control efficacy, followed by **C5**, **C7**, **C8**, **C11**, and **C12**. In the untreated tubers, **C1**, **C2**, **C6**, and **C10** strongly controlled EB infection while **C3** and **C4** were equal to the penthiopyrad in EB control. In the tuber assay, all compounds significantly controlled EB during preventive treatment as compared to curative and eradicant treatment.

**Table 4:** Comparison of lesion diameter and % disease (EB) control after treatment with SDHIs on potato tubers.

**Compounds**	**Treatment**					
	**Pre-treatment**		**Co-treatment**		**Post-treatment**	
	**Lesion diameter (mm)**	**Control efficacy**	**Lesion diameter (mm)**	**Control efficacy**	**Lesion diameter (mm)**	**Control efficacy**
DMSO	12.988 a (± 0.187)		13.828 a (± 0.246)		13.35 a (± 0.146)	
Penthiopyrad	3.470 b (± 0.056)	73.283	5.308 b (± 0.149)	61.614	5.891 b (± 0.134)	55.872
C1	3.153 cd (± 0.014)	75.723	3.745 d (± 0.157)	72.917	4.316 e (± 0.053)	67.67
C2	3.162 cd (± 0.018)	75.654	3.595 d (± 0.035)	74.002	4.349 e (± 0.045)	67.423
C3	3.391 bc (± 0.038)	73.891	4.507 c (± 0.167)	67.406	5.666 bcd (± 0.053)	57.558
C4	3.399 bc (± 0.042)	73.829	4.803 bc (± 0.199)	65.266	5.674 bcd (± 0.054)	57.498
C5	3.308 bcd (± 0.043)	74.303	4.728 bc (± 0.137)	65.808	5.720 bcd (± 0.046)	57.153
C6	3.191 bcd (± 0.03)	75.431	3.478 d (± 0.041)	74.848	4.241 e (± 0.078)	68.232
C7	3.299 bcd (± 0.051)	74.599	4.795 bc (± 0.24)	65.323	5.391 d (± 0.048)	59.617
C8	3.324 bcd (± 0.051)	74.407	4.807 bc (± 0.131)	65.237	5.498 cd (± 0.077)	58.816
C9	3.212 bcd (± 0.031)	75.277	5.016 bc (± 0.143)	63.725	5.537 bcd (± 0.061)	58.524
C10	3.082 d (± 0.016)	76.27	3.37 d (± 0.046)	75.629	4.307 e (± 0.062)	67.737
C11	3.228 bcd (± 0.036)	74.684	4.849 bc (± 0.129)	64.933	5.837 bc (± 0.061)	56.277
C12	3.262 bcd (± 0.027)	74.884	4.958 bc (± 0.162)	64.145	5.628 bcd (± 0.065)	57.842

*Values in parentheses indicate standard error. Means sharing the same letters are not significantly different (P < 0.05; LSD, Statistix 8.1)*.

When applied in co-treatment, the control efficacy of SDHIs was decreased. Among all the co-treated tubers, **C6** and **C10** treated tubers showed the lowest lesion sizes of 3.478 and 3.37 mm, respectively. Control efficacy of **C4**, **C5**, **C7**, **C8**, **C9**, **C11**, and **C12** was not significantly different from positive control while the least antifungal potential was exhibited by **C9** (lesion diameter of 5.016 cm) upon curative treatment.

During eradicant treatment, **C6** was the most potent at 24 h after inoculation, as compared to control (Table 4, Figure 6). Moreover **C1**, **C2**, and **C10** also showed remarkable suppression in lesion development, while **C4**, **C5**, **C11**, and **C12** showed similar results as the positive control. **C11** was the least effective in EB control.

The control efficacy values were significantly different in preventive, curative, and eradicant activity on tubers (Table 4). The lesion diameter values were significantly lower on pre-treated tubers as compared with curative and eradicant treated tubers, whereas no significant differences were detected for **C1**, **C6**, **C9**, and **C10** in pre-treated tubers. Comprehensively, compound **C1**, **C2**, **C6**, and **C10** showed high antifungal activity against dry rot of potato caused by *A. solani*.

### Enzyme assay

**Figure 7:**
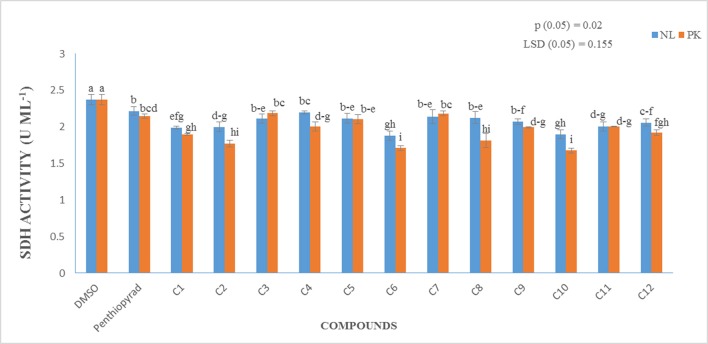
Measurement of Succinate Dehydrogenase Activity (*in vitro*) of 12 SDHIs.

A prime objective of this study was to compare the relative inhibitory potencies of twelve compounds on SDH activity of *A. solani*. Figure 7 show that all the compounds displayed inhibitory potencies toward *A. solani* SDH *in vitro*. However, **C1–C3**, **C6**, **C9–C11** showed the higher inhibitory activities against *A. solani* SDH. **C6** possesses the strongest inhibitory activity, (1.879 UmL^−1^), while **C4** possesses the lowest inhibitory activity (2.197 UmL^−1^). The results proved that SDHIs designed *in silico* displayed good inhibitory effects on *A. solani* SDH *in vitro*.

## Discussion

**Figure 8:**
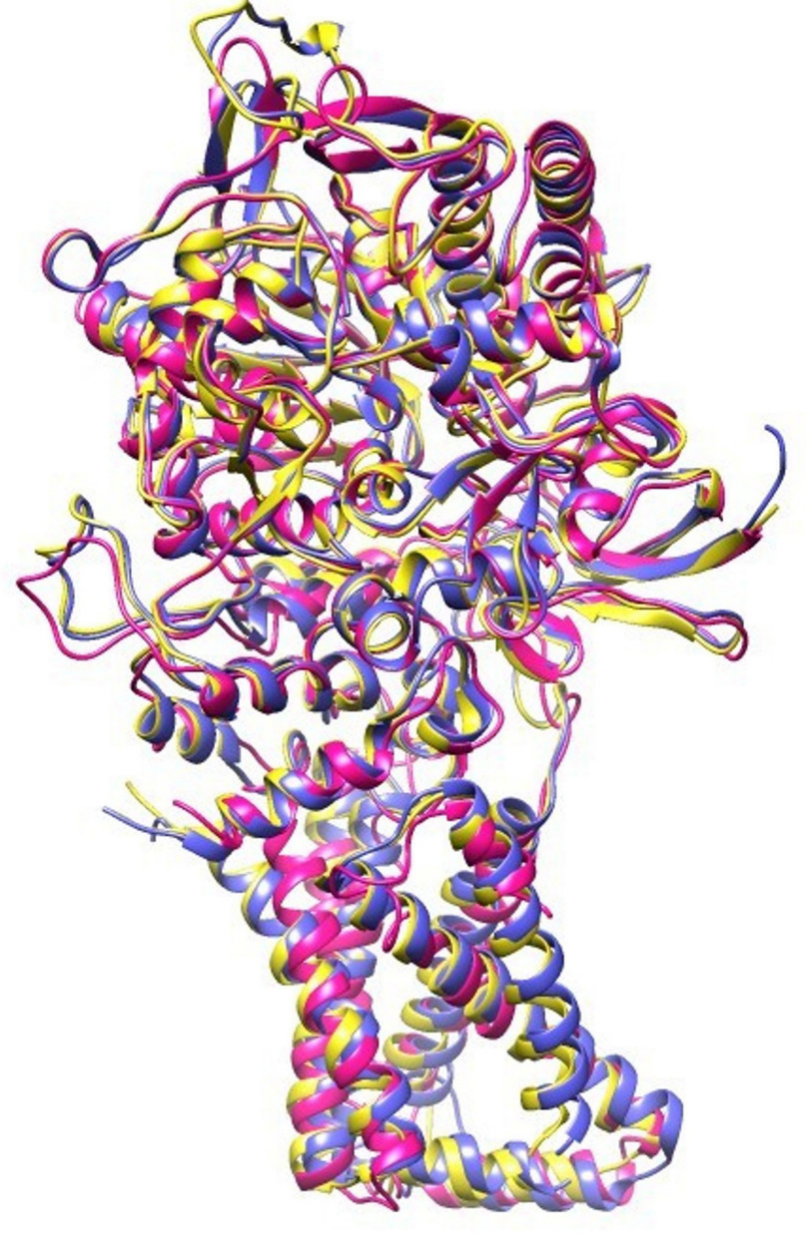
Superimposed view of *A. solani* SDH model (pink) with 1YQ3 (blue) and 1ZOY (yellow).

EB, caused by *A. solani*, is a ubiquitous disease that can reduce potato yield. This disease is usually controlled by fungicidal treatments (Pasche et al., 2005). Molecular modeling tools can be used to discover novel fungicides. In the present study, homology modeling of SDH was conducted. SDH is composed of a flavoprotein, an Ip, and a membrane-integral protein, which usually is a b-type cytochrome. The flavoprotein contains one covalently attached FAD and harbors the dicarboxylate binding site where succinate is oxidized to fumarate. The iron-sulfur protein contains three iron-sulfur clusters; a [2Fe-2S] (S-1), a [4Fe-4S] (S-2), and a [3Fe-4S] (S-3). In the *A. solani* SDH model, quinone forms a direct H-bond with Trp235 of SDHB and Tyr145 of SDHD. Arg76 of SDHC forms two H-bonds (2.27 and 1.80) with ubiquinone (Figure 1B). In a prior study, it was shown that the ubiquinone binding site is composed of SDHB, SDHC and SDHD, and is highly conserved between bacteria and eukaryotes (Sun et al., 2005). This is in agreement with Horsefield et al. (2006), who concluded that the ubiquinone binding site is a hydrophobic pocket formed by SDHB, C and D. The predicted 3D-model showed similar trans-membrane topology and secondary structural arrangement to the template. Multiple alignment of 1YQ3 and 1ZOY against the *A. solani* SDH shows that the residues forming the catalytic site and cofactor binding sites are well conserved. Several attempts were made to model the anchor subunits, however, the generated model was not of good quality based on Ramachandran plot, hence the anchor subunit was built by ITASSER (Wu et al., 2007). SDH of 1YQ3 and 1ZOY indicated homologies of 69% for SDHA and 69 and 67% for SDHB, respectively (Supplementary Table S1). The superimposed view of SDHA and SDHB of templates and the *A. solani* SDH model are shown in Figure 8. The RMSD between SDHA of model and templates (1YQ3 and 1ZOY) are 0.82 and 0.78, respectively. The RMSD values are 1.12 (1YQ3) and 1.09 (1ZOY) for SDHB. The homologies for chain C and D are 37 and 35%, respectively. The RMSD between SDHC of the model and 1YQ3 and 1ZOY are 1.18 and 1.16, respectively, while RMSD between chain D and 1YQ3 and 1ZOY are 1.12 and 1.26, respectively.

SBVS offers a good opportunity to discover selective SDH antagonists to treat EB disease. Here we report the application of a computational protocol combining SBVS, docking and scoring aimed to identify hits with previously untested molecular scaffolds as SDHIs. Our docking results revealed the importance of Trp69, Ser73, and Tyr145, which plays a vital role in protein-ligand complex formation and stabilization. In summary, the binding modes of these compounds reveal that most of the compounds form H-bond with Trp69, Ser73, or Tyr145, indicating the importance of these residues in the ubiquinone binding site of SDH. In the *E. coli* SQR structure, Tyr-83 is proposed to be the key residue for ubiquinone binding; and is a unique feature to SQR (Yankovskaya et al., 2003). The residues Tyr145, Arg76, Leu60, Ile77, Trp69, Trp234, Trp235, Tyr145, and Pro231, are conserved in templates 1ZOY and 1YQ3. These results are in accordance with the results observed by Shimizu et al. (2012), who reported that the binding site is surrounded by conserved residues (Ser72, and Arg76 of SDHC, Asp106 and Tyr107 of SDHD) and involved in H-bond networks with rhodoquinone (RQ) where RQ is H-bonded with Tyr107 (SDHD) and Ser72 (SDHC), also RQN forms an H-bond with Arg76 and Ser72 of SDHC (Shimizu et al., 2012). Our results also agree with available crystal structures and models of the transmembrane domain of complex II.

SDHIs are efficient fungicides that are widely used to control plant diseases caused by phytopathogenic fungi, although their effectiveness is undermined by the development of resistance across a range of different fungi. In a survey by Wharton in 2009, 15% of *A. solani* isolates were resistant to boscalid (Fairchild et al., 2012). The use of SDHI's has led to the selection of resistant strains in numerous pathogens in field conditions (Harrison et al., 1965; Harrison and Venette, 1970; Douglas and Groskopp, 1974; Bartlett et al., 2002). We evaluated the antifungal potential of 12 novel SDHIs against the EB pathogen, among them **C1**, **C6**, and **C10** were highly effective in reducing the radial growth of *A. solani in vitro*. The average disease control ranged from 69 to ≥81% for droplet inoculation of detached leafs and from ≥67 to ≥82% for the intact plants during preventive treatment. **C1**, **C2**, **C6**, and **C10** also showed potential to control disease in plants before inoculation. On the one hand, application before 24 hrs of inoculation is favorable for fungicide efficacy, because the spores are exposed to high fungicide concentrations which are not diluted by leaf growth or metabolism in the plant at the inoculation time point. On the other hand, the high number of spores and the optimal conditions to establish infection and develop disease result in a very high infection pressure, which challenges the fungicide performance. Plants sprayed 24 h after inoculation had larger lesions compared to the preventative treatment, ranging from 59 to 73%. Among the plants sprayed before and after inoculation, **C10** was the most efficient treatment in disease control, followed by **C1**, **C6**, and **C2**. These findings are consistent with other studies which show that the most effective control method is a protectant fungicide spray used in early growing season to vine kill (Harrison et al., 1965; Bartlett et al., 2002). In the *in vitro* SDH assay, **C6** and **C10** showed higher potency.

In this study, four compounds **C1**, **C2**, **C6**, and **C10** were found to be effective in control of *A. solani* in both *in vitro* and *in vivo* screening. We note here that the primary aim of using fungicides is effective disease control, and the choice of protective, curative or eradicant sprays or a combination thereof can have a major influence on the efficacy of disease control. To maintain effective control of disease, it is crucial to prevent the epidemic to be developed in the early season.

## Conclusion

In summary, we aimed to identify novel SDHIs through SBVS of a library of 1.7 million compounds. Initially, the three dimensional structure of *A. solani* SDH was modeled by homology modeling. Later on, 12 compounds were selected for biological testing based on docking results and binding interactions. The inhibitors target the quinone binding site and forms multiple interactions. The compounds showed excellent *in vitro* and *in vivo* results. Hence, the computational protocol led to the successful discovery of new *A. solani* inhibitors. We believe that these compounds can function as a starting point for the discovery of promising new fungicides candidates that can act as *A. solani* SDH inhibitors.

## Author contributions

This study was designed, directed and coordinated by AA-H, SH, and SI. SH provided conceptual and technical guidance for all the aspects of *in silico* studies. SI contributed in drafting the article, performed data collection, performed bioinformatics analyses, performed *in vitro* and *in vivo* experiments. PW helped with the design of the disease experiments, by providing technical assistance and also by giving feedback on the draft manuscript. VV provided research facilities for *in vitro* and *in vivo* experiments and revised the article critically. AK, AA-H and SA provided valuable suggestions in manuscript writing. SI and SH drafted and revised the article critically, and commented on by all authors.

### Conflict of interest statement

The authors declare that the research was conducted in the absence of any commercial or financial relationships that could be construed as a potential conflict of interest.

## Supplementary material

### Supporting information

Sequences of *A. solani* SDH subunits, Computational Work Flow, Ramachandran plot of SDHA, B, C, and D, and complete model, binding modes of 18 SDHIs, Multiple sequence alignment of SDHA, B, C, D, Pharmacophore model, Chemical structure and docking scores of 18 fungicides, chemical structures and docking score of 25 ZINC compounds and sequence identities between *A. solani* SDHs and templates are included in supporting information.
