# Supplementary material for: Discovering Novel Alternaria solani Succinate Dehydrogenase Inhibitors by in Silico Modeling and Virtual Screening Strategies to Combat Early Blight
Source: Front Chem. 2017 Nov 17;5:100. doi: 10.3389/fchem.2017.00100 (PMC5698277; doi:10.3389/fchem.2017.00100)
Supplement: Supplementary file 1 [file Presentation1.pdf]

Supporting Information

Discovering Novel *Alternaria solani* Succinate Dehydrogenase Inhibitors by *In Silico* Modeling and Virtual Screening Strategies to Combat Early Blight

Sehrish Iftikhar[1], Ahmad Ali Shahid[1,2], Sobia Ahsan Halim[3,*], Jaap Wolters[4], Vivianne G A A Vleeshouwers[4], Ajmal Khan[5,6,*], Ahmed Al-Harrasi[6], Shahbaz Ahmad[1]

[1]Institute of Agricultural Sciences, University of the Punjab, Lahore, Pakistan. [2]Center of Excellence in Molecular Biology, University of the Punjab, Lahore, Pakistan. [3]Department of Biochemistry, Kinnaird College for Women, 93-Jail Road, Lahore-Pakistan. [4]Wageningen UR Plant Breeding, Wageningen University and Research Center, Wageningen, The Netherlands. [5]Department of Chemistry, COMSATS Institute of Information Technology, Abbottabad-22060, Pakistan. [6]UoN Chair of Oman Medicinal Plants and Marine Products, University of Nizwa, Sultanate of Oman

***Correspondence:** Sobia Ahsan Halim, E-mail: sobia.ahsan@kinnaird.edu.pk sobiahal@gmail.com, Tel. Off: +(92) 42 99203781-84 (Ext 226), Ajmal Khan, email: ajmalchemist@yahoo.com

Supplementary Data

Sequences of *A. solani* SDH subunits.

***A. solani* SDH subunit A**
PVIDHEYDAIVVGAGGAGLRAAFGLAEAGFNTACISKLFPTRSHTVAAQGGINAALGSM
HEDDWRWHMYDTVKGSDWLGDQDAIHYMTREAPQSVIELENYGCPFSRTDDGKMYQ
RAFGGQSQKYGKGGQAYRCCAAADRTGHALLHTLYGQSLRHNTKYFIEFFATDLIMED
GVCKGVVAYNQEDGTIHRFIAKNTVLATGGYGRAYFSCTSAHTCTGDGMAMVARAGL
PNQDLEFVQFHPTGIYGAGCLITEGSRGEGGYLLNSEGERFMERYAPTAKDLASRDVVS
RSMTLEIREGRGVGPEKDHIYLQLSHLPAEVLHERLPGISETAAIFAGVDVTKQPIPVLPT
VHYNMGGIPTKYTGEVITQDAQGNDQVVPGLFACGEAASVSVHGANRLGANSLLDLIV
FGRAVSHTIRDNFSPGQKADPVSADAGADSISVLDQIRTSDGPKSTAEIRLQMQKVMQT
DVAVFRTQESLDEGVKKIHDVDADFANVGIKDRSMIWNSDLVETLELRNLLTCAVQTA
ESAANRKESRGAHAREDFPDRDDEQWMKHTLSWQKKPHGETQLGYRKVVGTTLDEAE
CKAVPPFKRTY

***A. solani* SDH subunits B (GenBank: AGS56260.1)**
MASIRAFTRLATSDLVPVRPAVFSRGFASVNDVHAREPISKIAEKIAPDASRSPVPESKTS
TVPEPEPSKDAKTKTFHIYRWNPDEPTSKPKMQSYTLDLNKTGPMMLDALIRIKNEVDP
TLTFRRSCREGICGSCAMNIDGVNTLACLCRIPTDTTKESRIYPLPHMYVVKDLVPDMTL
FYKQYRSVKPYLQRSTAAPDGREFRQSKEDRKKLDGLYECILCACCSTSCPSYWWNQE
EYLGPAVLLQSYRWIADSRDEKKAERQDALNNSMSLYRCRTILNCSRTCPKGLNPALAI
AEIKKSMAFT

***A. solani* SDH subunits C (AGS56262.1)**
MASQRVFQLGLRRAAAPGLRVQPVGRMVQRRLAATEHASQSEAAEILAKQRINRPVSP
HLAIYRPQITWYASSLNRITGITLSGSLYLFGIAYLIAPYTGWHMETQSMVATVAAWPAA
AKAGLKAFYAFPFFFHSFNGLRHLSWDVGIGFKNQQVIRLGW

***A. solani* SDH subunits D (GenBank: AGS56264.1)**

MASVMRPGLLRQACPPAHASQRMLSTATATTSRPLAQQLRPAFQRSAVPKATRIAAFH
ATQRTQILPPLPQKIIGTTNDPVPVPDPDYAHGSYHWSFERIVSAGLIPLTIAPFAAGSLNP
LTDSILCALLVVHSHIGFESCIIDYFPAKRVPKTRKAAMWALRAGTLTLGLALYSFETND
VGITE

Supplementary Figures and Tables

Supplementary Figures

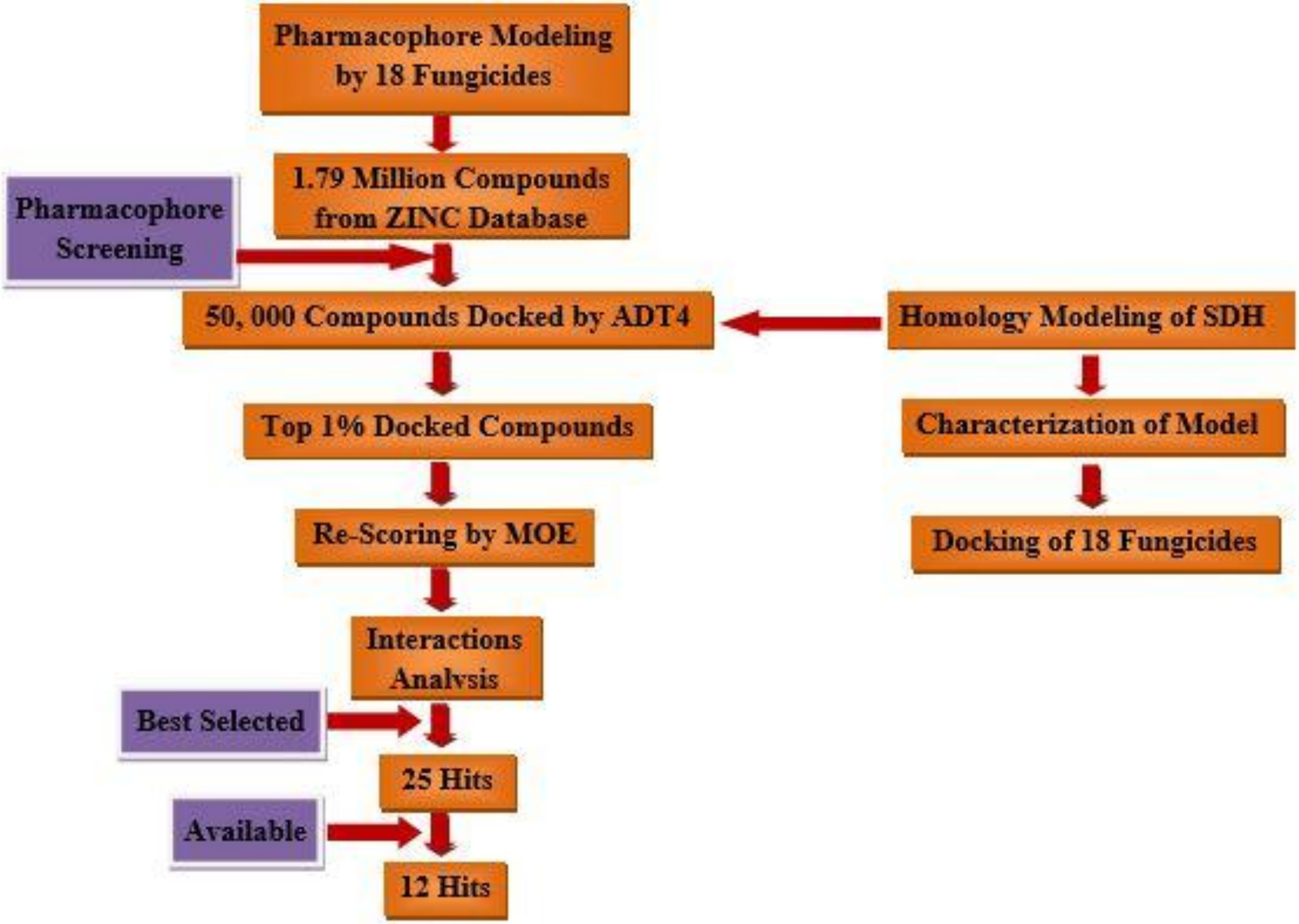

Supplementary Figure S1. Schematic presentation of Computational Work Flow

```
SDHA       -------------PVIDHEYDAIVVGAGGAGLRAAFGLAEAGFNTACISKLFPTRSHTVA
1YQ3A      -STKVSDSISTQYPVVDHEFDAVVVGAGGAGLRAAFGLSEAGFNTACVTKLFPTRSHTVA
1ZOYA      SSAKVSDAISTQYPVVDHEFDAVVVGAGGAGLRAAFGLSEAGFNTACVTKLFPTRSHTVA
                        **:***:**:***************:********::***********

SDHA       AQGGINAALGSMHEDDWRWHMYDTVKGSDWLGDQDAIHYMTREAPQSVIELENYGCPFSR
1YQ3A      AQGGINAALGNMEDDNWRWHFYDTVKGSDWLGDQDAIHYMTEQAPAAVIELENYGMPFSR
1ZOYA      AQGGINAALGNMEEDNWRWHFYDTVKGSDWLGDQDAIHYMTEQAPASVVELENYGMPFSR
           **********.*.:*:****:********************.:** :*:****** ****

SDHA       TDDGKMYQRAFGGQSQKYGKGGQAYRCCAAADRTGHALLHTLYGQSLRHNTKYFIEFFAT
1YQ3A      TEEGKIYQRAFGGQSLQFGKGGQAHRCCCVADRTGHSLLHTLYGRSLRYDTSYFVEYFAL
1ZOYA      TEDGKIYQRAFGGQSLKFGKGGQAHRCCCVADRTGHSLLHTLYGRSLRYDTSYFVEYFAL
           *::**:********* ::******:***..******:*******:***::*.**:*:**

SDHA       DLIMEDGVCKGVVAYNQEDGTIHRFIAKNTVLATGGYGRAYFSCTSAHTCTGDGMAMVAR
1YQ3A      DLLMENGECRGVIALCIEDGTIHRFRAKNTVIATGGYGRTYFSCTSAHTSTGDGTAMVTR
1ZOYA      DLLMENGECRGVIALCIEDGSIHRIRARNTVVATGGYGRTYFSCTSAHTSTGDGTAMVTR
           **:**:* *:**:*   ***:***: *:***:*******:*********.**** ***:*

SDHA       AGLPNQDLEFVQFHPTGIYGAGCLITEGSRGEGGYLLNSEGERFMERYAPTAKDLASRDV
1YQ3A      AGLPCQDLEFVQFHPTGIYGAGCLITEGCRGEGGILINSQGERFMERYAPVAKDLASRDV
1ZOYA      AGLPCQDLEFVQFHPTGIYGAGCLITEGCRGEGGILINSQGERFMERYAPVAKDLASRDV
           **** ***********************.***** *:**:**********.*********

SDHA       VSRSMTLEIREGRGVGPEKDHIYLQLSHLPAEVLHERLPGISETAAIFAGVDVTKQPIPV
1YQ3A      VSRSMTIEIREGRGCGPEKDHVYLQLHHLPPQQLATRLPGISETAMIFAGVDVTKEPIPV
1ZOYA      VSRSMTLEIREGRGCGPEKDHVYLQLHHLPPEQLAVRLPGISETAMIFAGVDVTKEPIPV
           ******:******* ******:**** *** : *  ********* *********:****

SDHA       LPTVHYNMGGIPTKYTGEVITQDAQGNDQVVPGLFACGEAASVSVHGANRLGANSLLDLI
1YQ3A      LPTVHYNMGGIPTNYKGQVITH-VNGEDKVVPGLYACGEAASASVHGANRLGANSLLDLV
1ZOYA      LPTVHYNMGGIPTNYKGQVLRH-VNGQDQVVPGLYACGEAACASVHGANRLGANSLLDLV
           *************:*.*:*: : .:*:*:*****:******..****************:

SDHA       VFGRAVSHTIRDNFSPGQKADPVSADAGADSISVLDQIRTSDGPKSTAEIRLQMQKVMQT
1YQ3A      VFGRACALTIAETCKPGEPVPSIKPNAGEESVANLDKLRFADGTIRTSEARLNMQKTMQS
1ZOYA      VFGRACALSIAESCRPGDKVPSIKPNAGEESVMNLDKLRFANGTIRTSELRLSMQKSMQS
           ***** : :* :.  **: .  :. :** :*:  **::* ::*   *:* **.*** **:

SDHA       DVAVFRTQESLDEGVKKIHDVDADFANVGIKDRSMIWNSDLVETLELRNLLTCAVQTAES
1YQ3A      HAAVFRTGSILQEGCEKLSQIYRDLAHLKTFDRGIVWNTDLVETLELQNLMLCALQTIYG
1ZOYA      HAAVFRVGSVLQEGCEKILRLYGDLQHLKTFDRGMVWNTDLVETLELQNLMLCALQTIYG
           ..****. . *:** :*:  :  *: ::   **.::**:********:**: **:**  .

SDHA       AANRKESRGAHAREDFPDRDD-----------------EQWMKHTLSWQKKPHGETQLGY
1YQ3A      AEARKESRGAHAREDYKLRIDEFDYSKPLQGQQKRPFEEHWRKHTLSYVDVKSGKVTLKY
1ZOYA      AEARKESRGAHAREDFKERVDEYDYSKPIQGQQKKPFQEHWRKHTLSYVDVKTGKVSLEY
           *  ************:  * *                 *:* *****: .   *:. * *

SDHA       RKVVGTTLDEAECKAVPPFKRTY
1YQ3A      RPVIDRTLNEEDCSSVPPAIRSY
1ZOYA      RPVIDKTLNEADCATVPPAIRSY
           * *:. **:* :* :***  *:*
```

Supplementary Figure S2 (A). Multiple sequence alignment of SDHA.

```
SDHB          MASIRAFTRLATSDLVPVRPAVFSRGFASVNDVHAREPISKIAEKIAPDASRSPVPESKT
1YQ3B         ------------------------------------------------------------
1ZOYB         ------------------------------------------------------------


SDHB          STVPEPEPSKDAKTKTFHIYRWNPDEPTSKPKMQSYTLDLNKTGPMMLDALIRIKNEVDP
1YQ3B         ---AQTAAAATSRIKKFSIYRWDPDKPGDKPRMQTYEVDLNKCGPMVLDALIKIKNELDS
1ZOYB         ---AQTAAATAPRIKKFAIYRWDPDKTGDKPHMQTYEIDLNNCGPMVLDALIKIKNEIDS
                  :   :   : *.* ****:**:  .**:**:* :***: ***:*****:****:*

SDHB          TLTFRRSCREGICGSCAMNIDGVNTLACLCRIPTDTTKESRIYPLPHMYVVKDLVPDMTL
1YQ3B         TLTFRRSCREGICGSCAMNIAGGNTLACTKKIDPDLSKTTKIYPLPHMYVVKDLVPDLSN
1ZOYB         TLTFRRSCREGICGSCAMNINGGNTLACTRRIDTNLDKVSKIYPLPHMYVIKDLVPDLSN
              ******************** * *****  :*  :  * ::*********:******::

SDHB          FYKQYRSVKPYLQRSTAAPDGR-EFRQSKEDRKKLDGLYECILCACCSTSCPSYWWNQEE
1YQ3B         FYAQYKSIEPYLKKKDESKQGKEQYLQSIEDRQKLDGLYECILCACCSTSCPSYWWNGDK
1ZOYB         FYAQYKSIEPYLKKKDESQEGKQQYLQSIEEREKLDGLYECILCACCSTSCPSYWWNGDK
              ** **:*::***::.  : :*: :: ** *:*:************************ ::

SDHB          YLGPAVLLQSYRWIADSRDEKKAERQDALNNSMSLYRCRTILNCSRTCPKGLNPALAIAE
1YQ3B         YLGPAVLMQAYRWMIDSRDDYTEERLAQLQDPFSLYRCHTIMNCTRTCPKGLNPGKAIAE
1ZOYB         YLGPAVLMQAYRWMIDSRDDFTEERLAKLQDPFSLYRCHTIMNCTGTCPKGLNPGKAIAE
              *******:*:***: ****: . **   *:: :*****:**:**: ********. ****

SDHB          IKKSMAFT-------
1YQ3B         IKKMMATYKEKAAAA
1ZOYB         IKKMMATYKEKKASV
              *** **
```

Supplementary Figure S2 (B). Multiple sequence alignment of SDHB.

```
SDHC          MASQRVFQLGLRRAAAPGLRVQPVGRMVQRRLAATEHASQSEAAEILAKQRINRPVSPHL
1YQ3C         --------------------MATTAKEEMARFWEKNTKS-------------SRPLSPHI
1ZOYC         --------------------LGTTAKEEMERFWNKNLGS-------------NRPLSPHI
                                  :  ..:    *:  .:  *             .**:***:

SDHC          AIYRPQITWYASSLNRITGITLSGSLYLFGIAYLIAPYTGWHMETQSMVATVAAWPAAAK
1YQ3C         SIYKWSLPMAMSITHRGTGVALSLGVSLFSLAALLLPEQ---FPHYVAVVKSLSLSPALI
1ZOYC         TIYRWSLPMAMSICHRGTGIALSAGVSLFGLSALLLPGN---FESHLELVKSLCLGPTLI
              :**: .:    *  :* **::** .: **.:: *: *     :     :..  .   :

SDHC          AGLKAFYAFPFFFHSFNGLRHLSWDVGIGFKNQQVIRLGW-----------------
1YQ3C         YSAKFALVFPLSYHTWNGIRHLVWDMGKGFKLSQVEQSGVVVLILTLLSSAGIAAIS
1ZOYC         YTAKFGIVFPLMYHTWNGIRHLIWDLGKGLTIPQLTQSGVVVLILTVLSSVGLAAM-
                 *   .**: :*::**:*** **:* *:.  *: : *
```

Supplementary Figure S2 (C). Multiple sequence alignment of SDHC.

```
SDHD        MASVMRPGLLRQACPPAHASQRMLSTATATTSRPLAQQLRPAFQRSAVPKATRIAAFHAT
1ZOYD       ------------------------------------------------------------
1YQ3D       ------------------------------------------------------------


SDHD        QRTQILPPLPQKIIGTTNDPVPVPDPDYAHGSYHWSFERIVSAGLIPLTIAPFAAGSLNP
1ZOYD       -------------------------ASSKAASLHWTGERVVSVLLLGLLPAAYLN--PCS
1YQ3D       -------------------------GSSKAASLHWTSERAVSALLLGLLPAAYLY--PGP
                                      .   .* **: ** **. *: *  * :

SDHD        LTDSILCALLVVHSHIGFESCIIDYFPAKRVPKTRKAAMWALRAGTLTLGLALYSFETND
1ZOYD       AMDYSLAAALTLHGHWGIGQVVTDYVRGDALQKAAKAGLLALSAFTFAG---LCYFNYHD
1YQ3D       AVDYSLAAALTLHGHWGLGQVITDYVHGDTPIKVANTGLYVLSAITFTG---LCYFNYYD
              *  *.* *.:*.* *: . : **. ..   *. ::.: .* * *::    *  *:  *

SDHD        VGITE--------
1ZOYD       VGICKAVAMLWKL
1YQ3D       VGICKAVAMLWSI
            *** :
```

Supplementary Figure S2 (D). Multiple sequence alignment of SDHD.

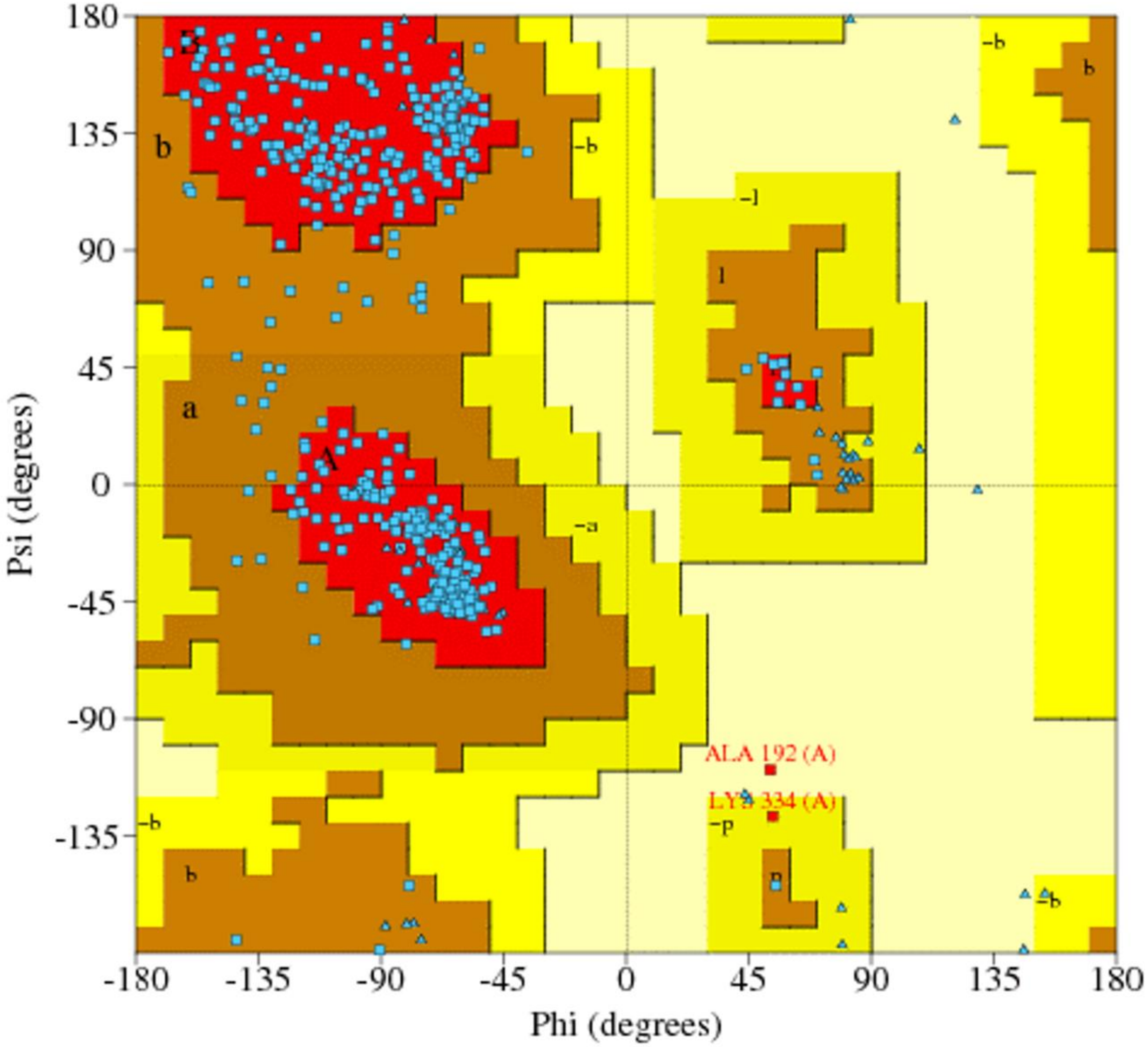

Ramachandran Plot statistics

		No. of residues	%-tage
Most favored regions	[A, B, L]	471	91.3%
Additional allowed regions	[a,b,l,p]	43	8.3%
Generously allowed regions	[~a,~b,~l,~p]	1	0.2%
Disallowed regions	[XX]	1	0.2%*****
Non-glycine and non-proline residues		516	100.0%
End-residues (excl. Gly and Pro)		1	
Glycine residues		61	
Proline residues		22	
Total number of residues		600	

Supplementary Figure S3 (A). Ramachandran plot of Subunit A

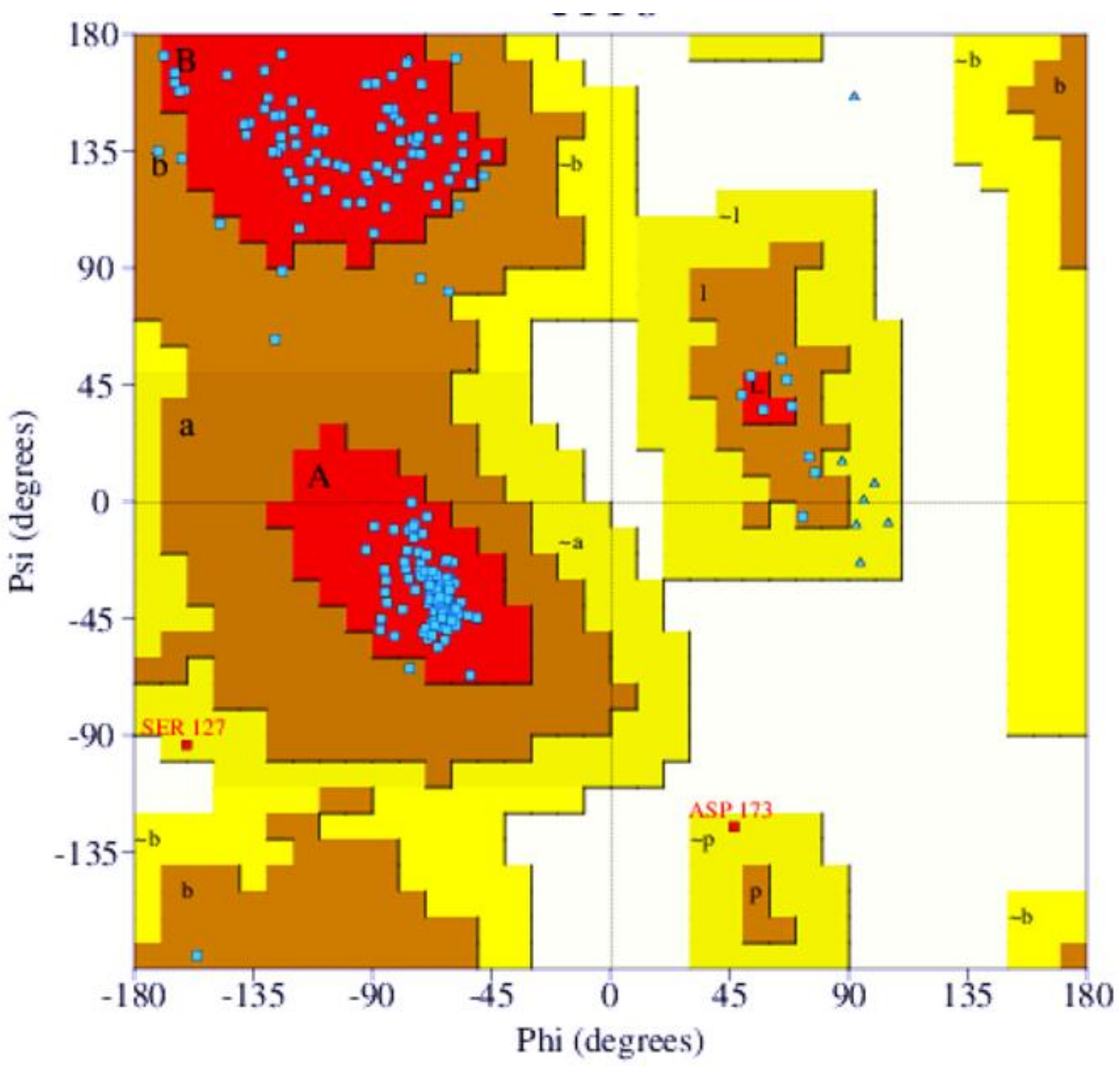

Ramachandran Plot statistics

		No. of residues	%-tage
Most favored regions	[A,B,L]	191	91.0%
Additional allowed regions	[a,b,l,p]	17	8.1%
Generously allowed regions	[~a,~b,~l,~p]	2	1.0%
Disallowed regions	[XX]	0	0.0%
Non-glycine and non-proline residues		210	100.0%
End-residues (excl. Gly and Pro)		2	
Glycine residues		8	
Proline residues		15	
Total number of residues		235	

Supplementary Figure S3 (B). Ramachandran plot of Subunit B

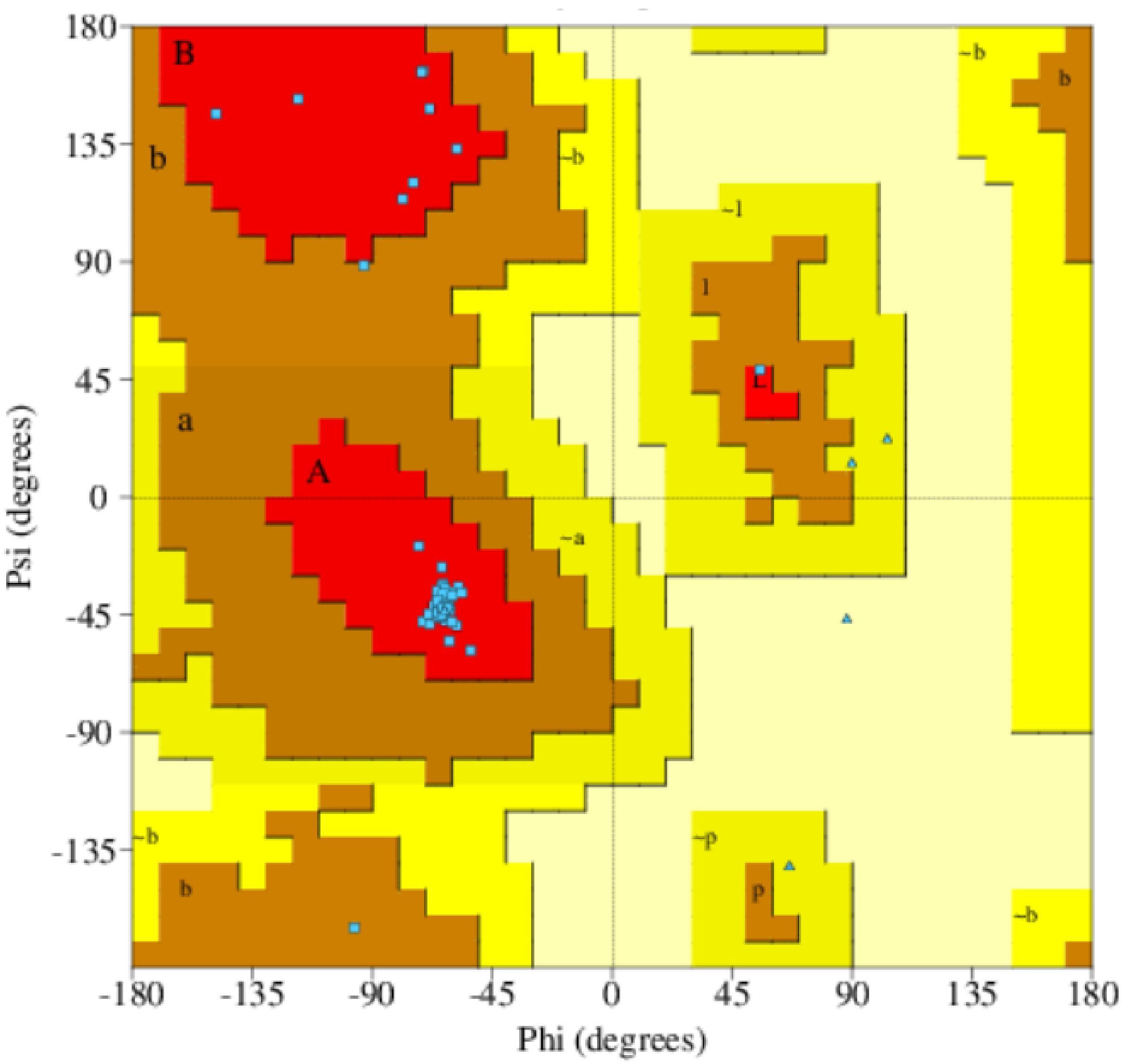

Ramachandran Plot statistics

	**No. of residues**	**%-tage**
Most favored regions [A,B,L]	78	97.5%
Additional allowed regions [a,b,l,p]	2	2.5%
Generously allowed regions [~a,~b,~l,~p]	0	0.0%
Disallowed regions [XX]	0	0.0%
Non-glycine and non-proline residues	80	100.0%
End-residues (excl. Gly and Pro)	2	
Glycine residues	9	
Proline residues	3	
Total number of residues	94	

Supplementary Figure S3 (C). Ramachandran plot of Subunit C

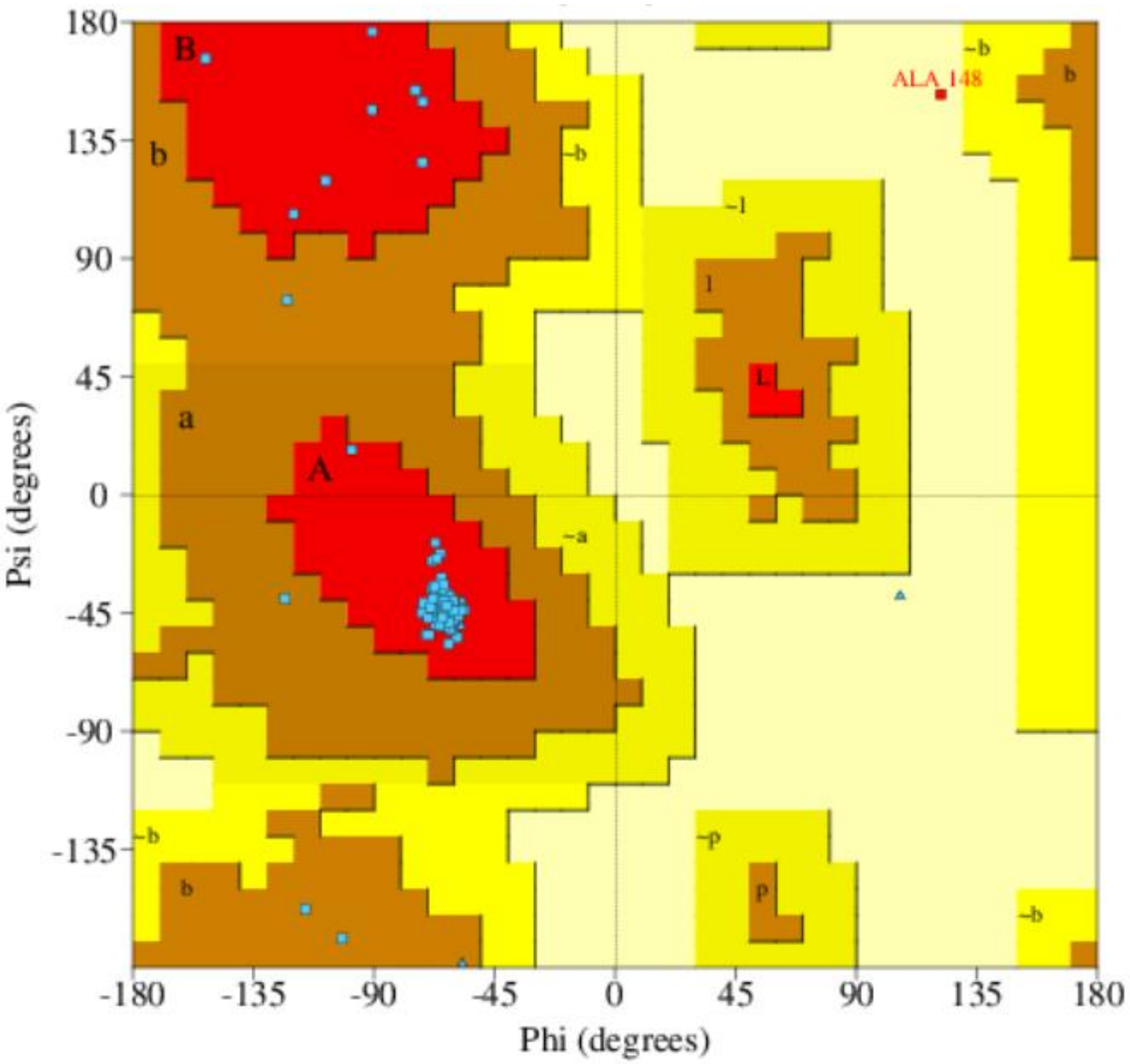

Ramachandran Plot statistics

		No. of residues	%-tage
Most favoured regions	[A,B,L]	81	94.2%
Additional allowed regions	[a,b,l,p]	4	4.7%
Generously allowed regions	[~a,~b,~l,~p]	0	0.0%
Disallowed regions	[XX]	1	1.2%*****
Non-glycine and non-proline residues		86	100.0%
End-residues (excl. Gly and Pro)		2	
Glycine residues		7	
Proline residues		6	
Total number of residues		101	

Supplementary Figure S3 (D). Ramachandran plot of Subunit D

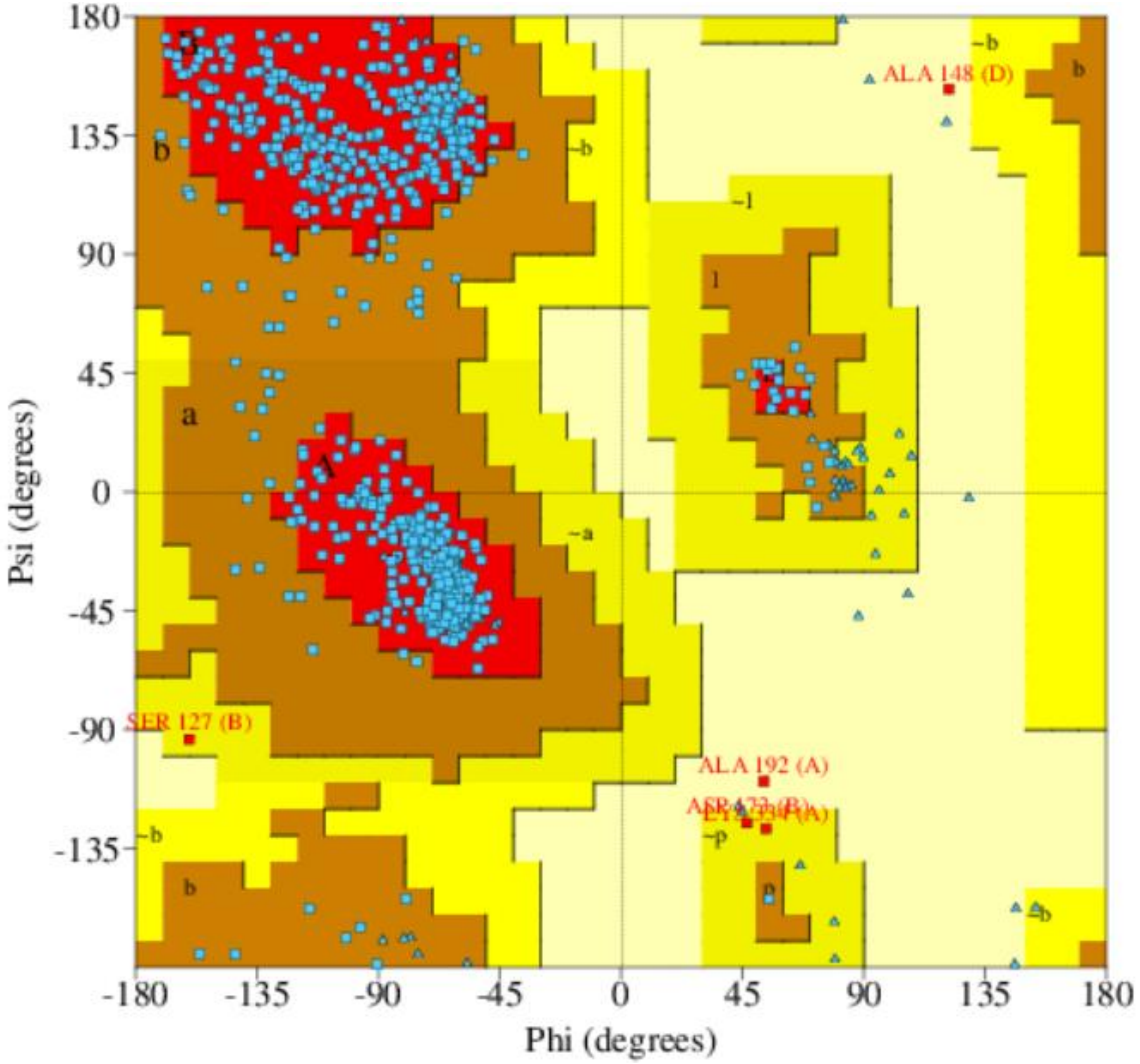

Ramachandran Plot statistics

	No. of residues	%-tage
Most favoured regions [A,B,L]	821	92.0%
Additional allowed regions [a,b,l,p]	66	7.4%
Generously allowed regions [~a,~b,~l,~p]	3	0.3%
Disallowed regions [XX]	2	0.2%*
Non-glycine and non-proline residues	892	100.0%
End-residues (excl. Gly and Pro)	7	
Glycine residues	85	
Proline residues	46	
Total number of residues	1030	

Supplementary Figure S3 (E). Ramachandran plot of complete model of SDH

Program: ERRAT2
File: /var/www/SAVES/Jobs/2076096//errat.pdb
Chain#:1
Overall quality factor**: 82.010

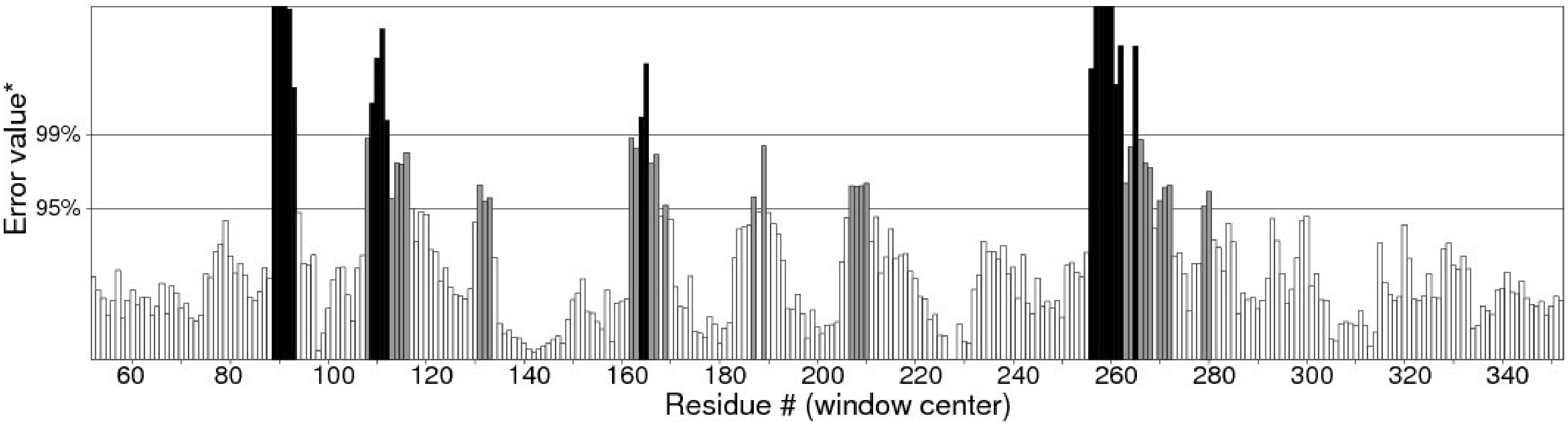

*On the error axis, two lines are drawn to indicate the confidence with which it is possible to reject regions that exceed that error value.

**Expressed as the percentage of the protein for which the calculated error value falls below the 95% rejection limit. Good high resolution structures generally produce values around 95% or higher. For lower resolutions (2.5 to 3A) the average overall quality factor is around 91%.

Supplementary Figure S4. ERRAT scores for the *A. solani* SDH model

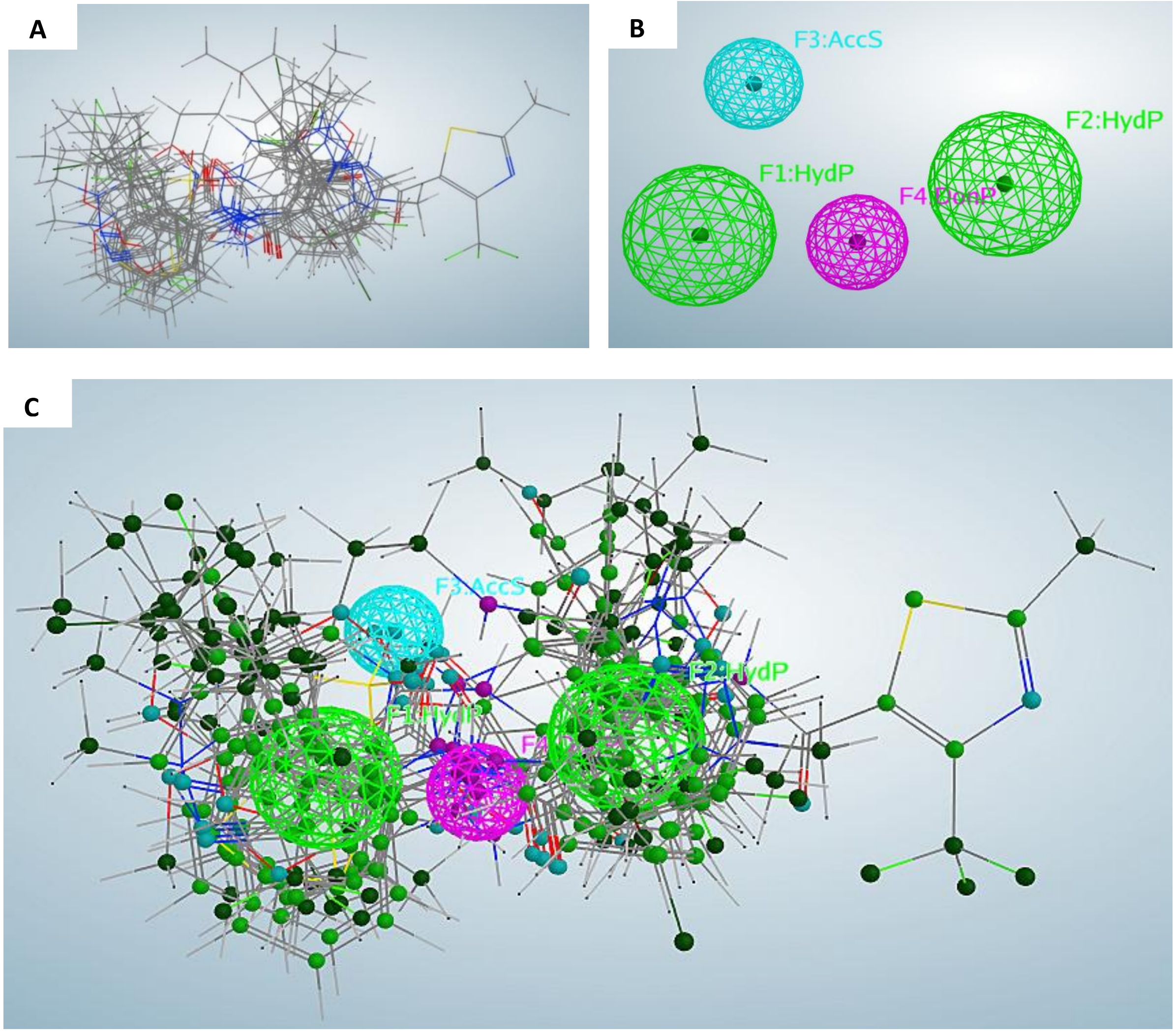

Supplementary Figure S5. (A) Superimposed view of 18 Fungicides (B) Pharmacophore model established by 18 Fungicides (SDH inhibitors). The pharmacophoric features H-bond acceptor, H-bond donor, and Hydrophobic features are depicted in light blue, pink, and Green spheres, respectively (C) Superimposed view of 18 fungicides on the pharmacophore model.

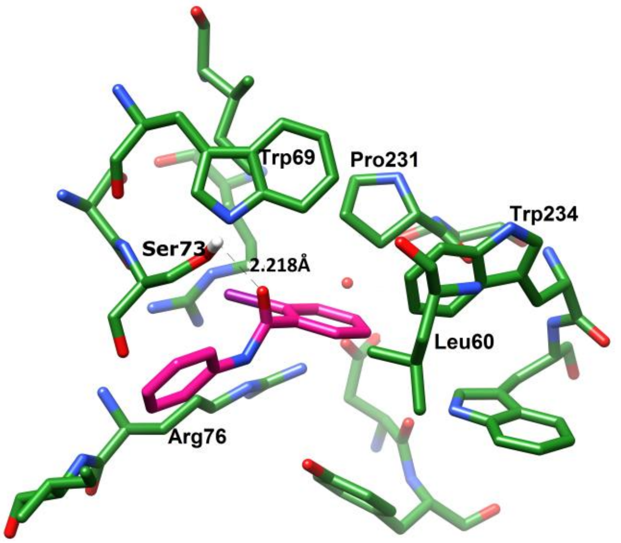

Benodanil

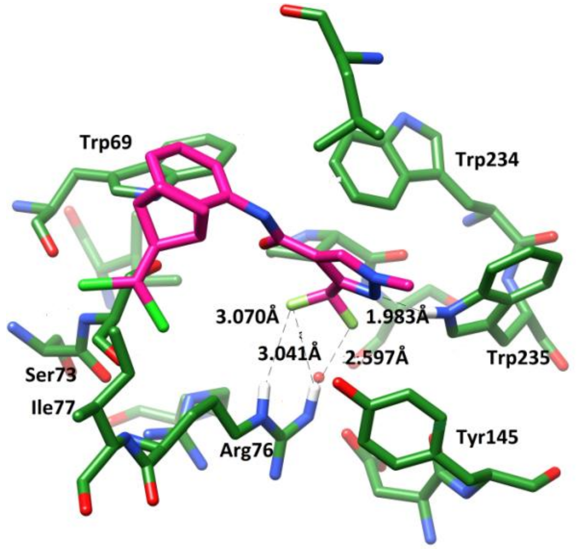

Benzovindiflupyr

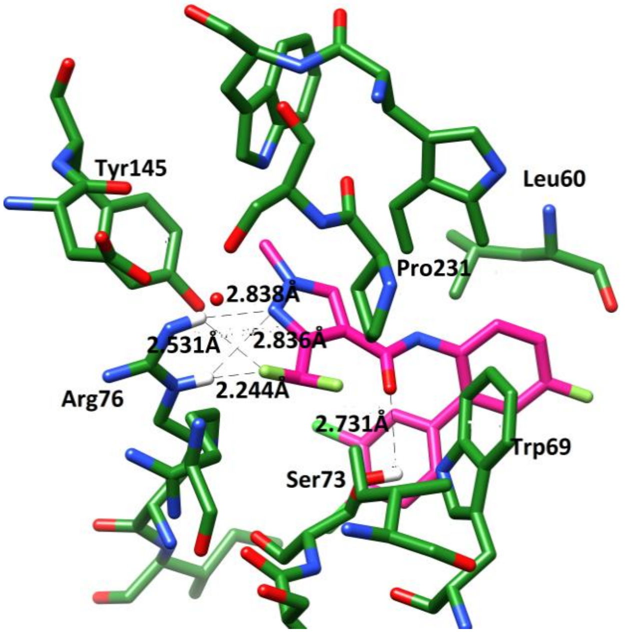

Bixafen

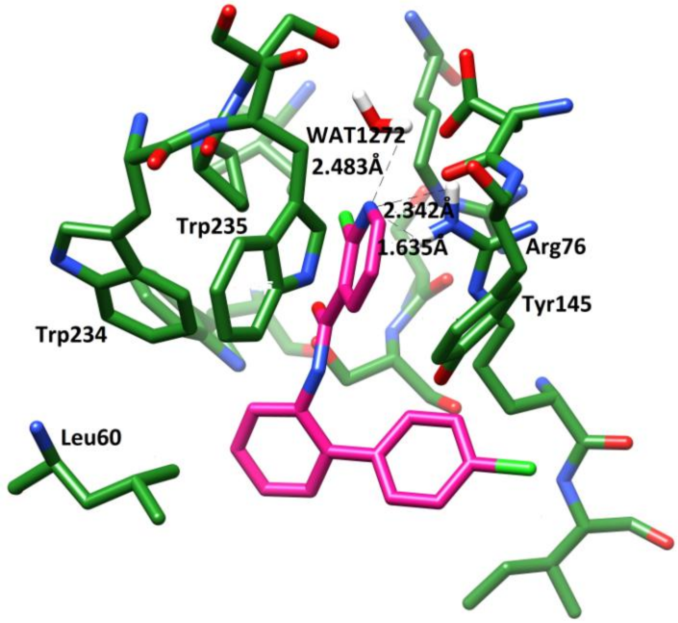

Boscalid

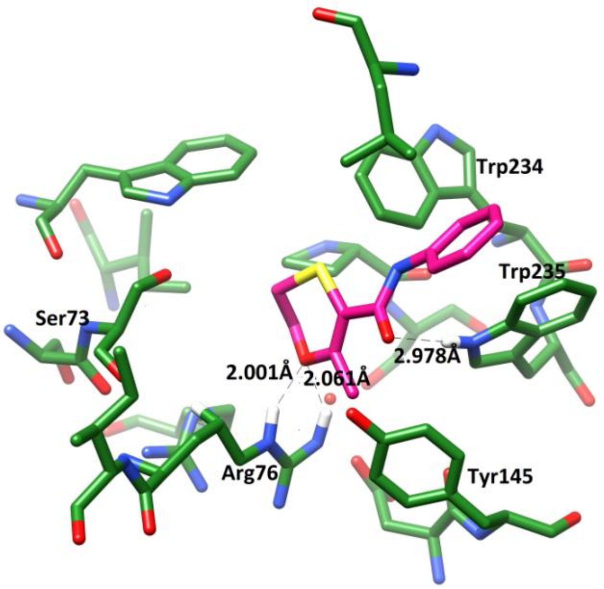

Carboxin

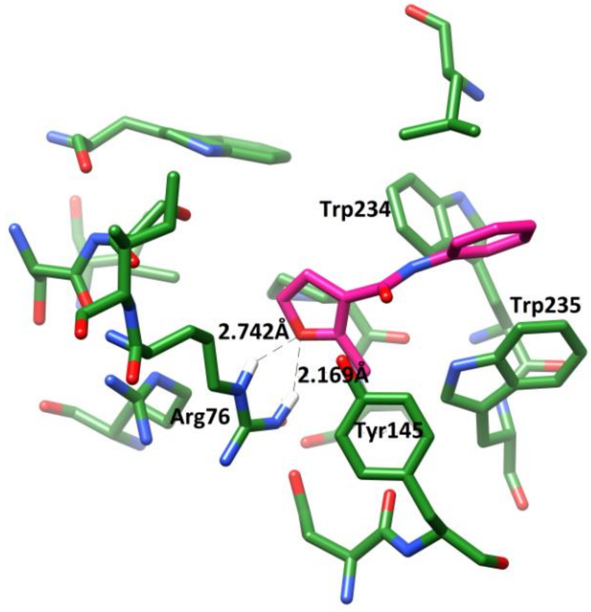

Fenfuram

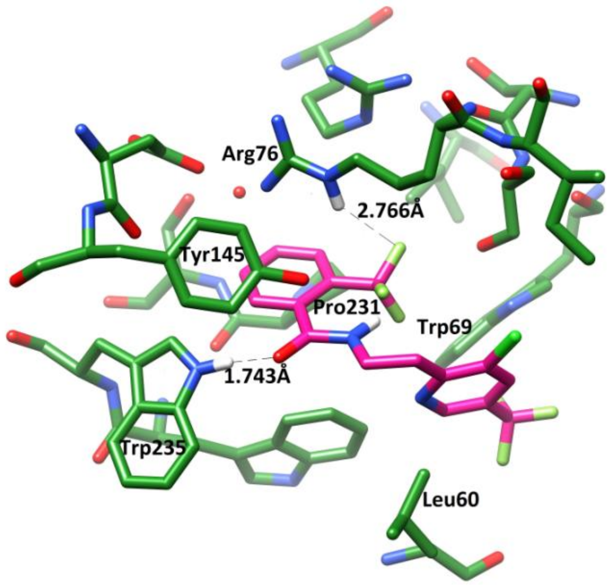

Fluopyram

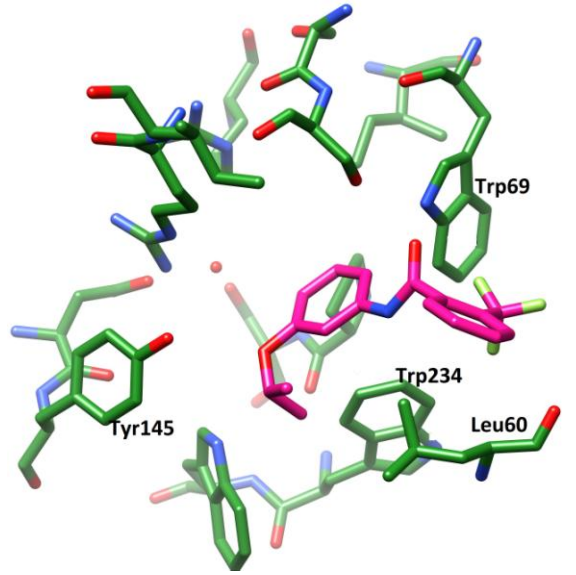

Flutolanil

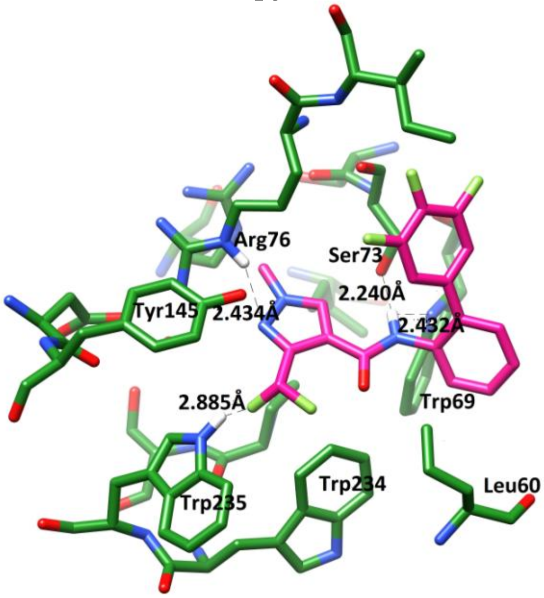

Fluxapyroxad

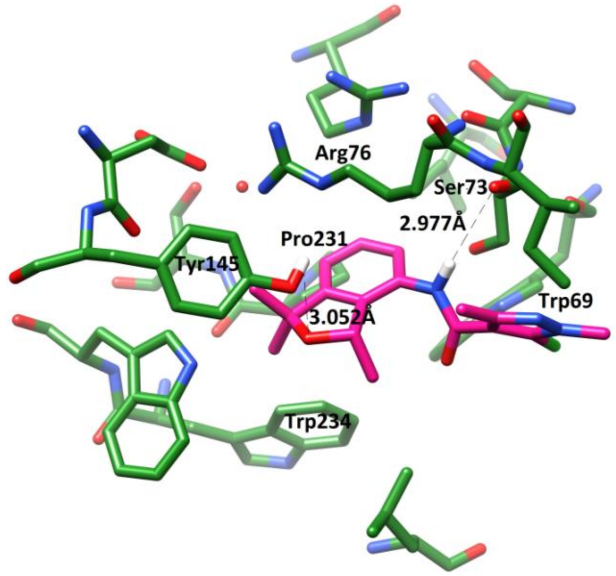

Furametpyr

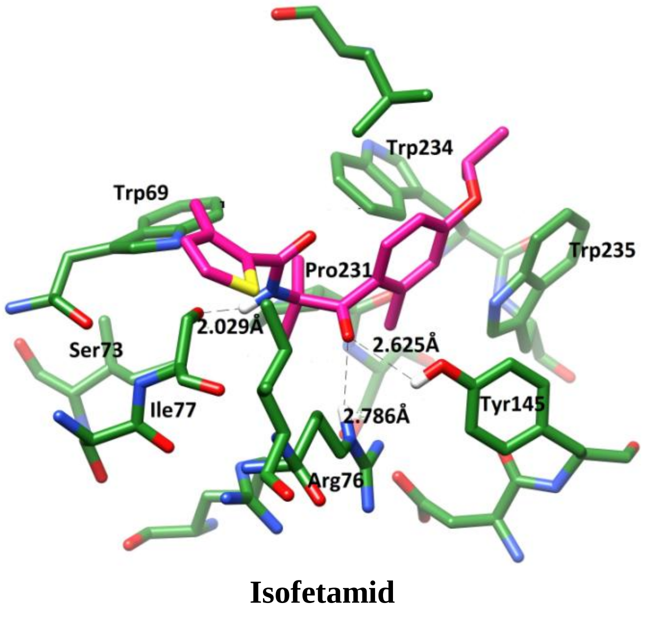

Isofetamid

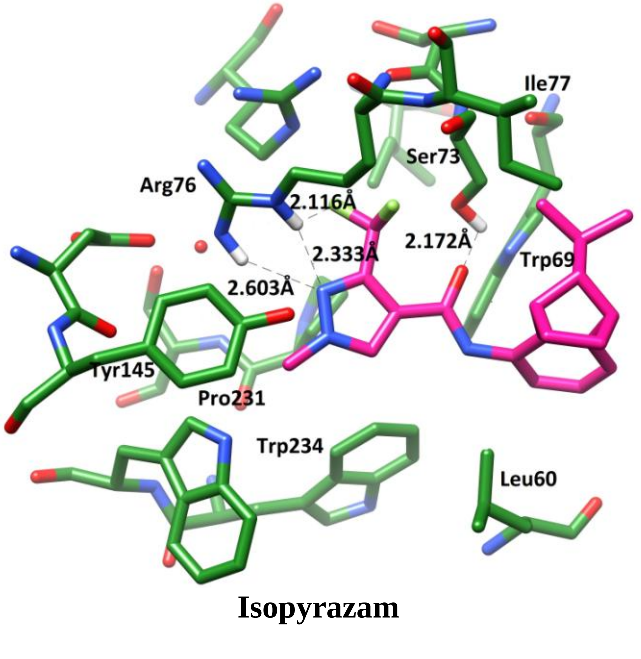

Isopyrazam

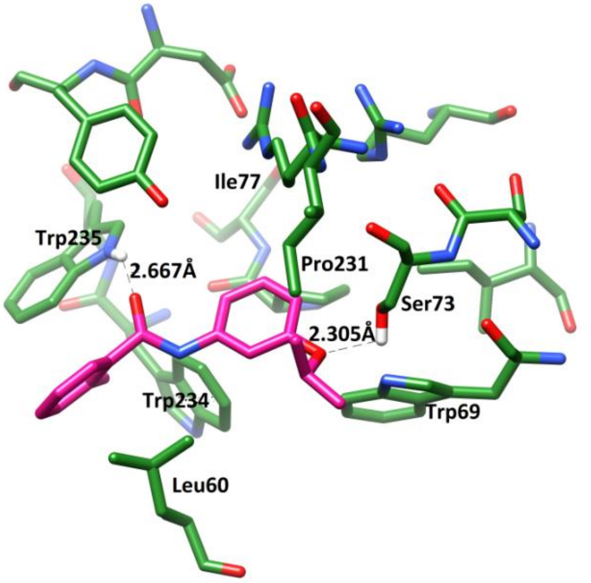

Mepronil

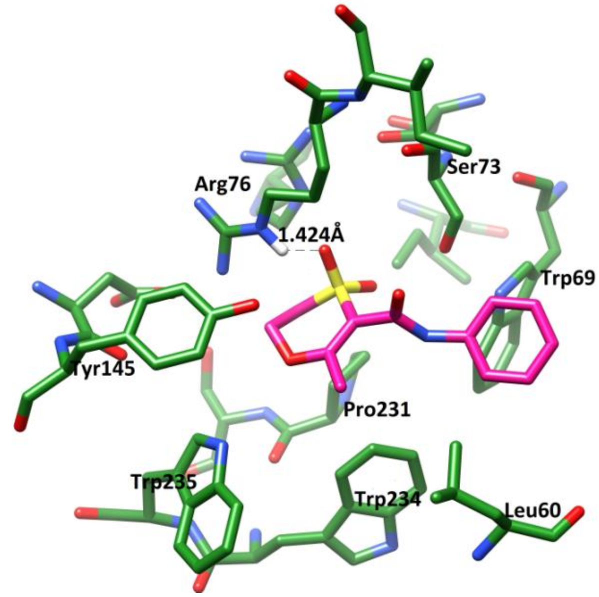

Oxycarboxin

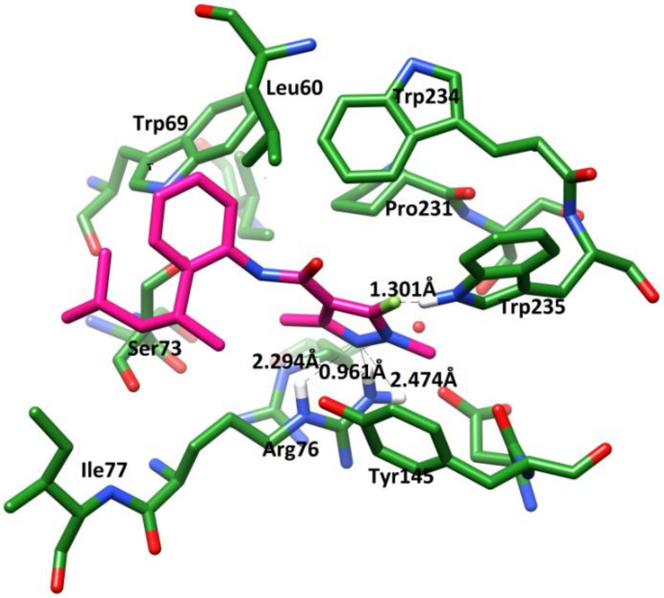

Penflufen

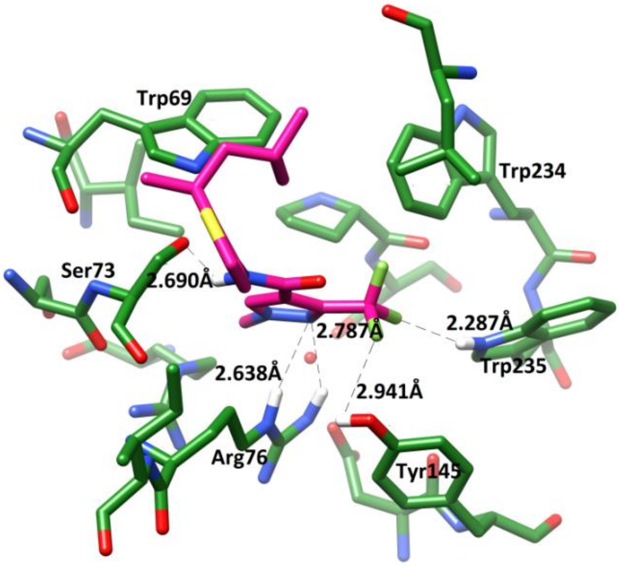

Penthiopyrad

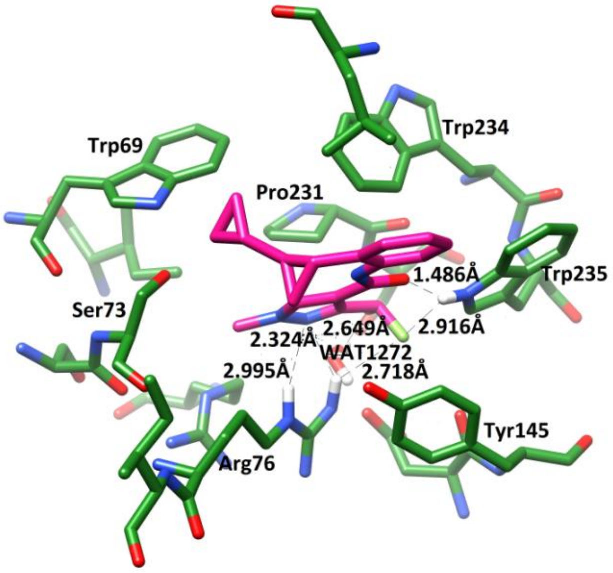

Sedaxane

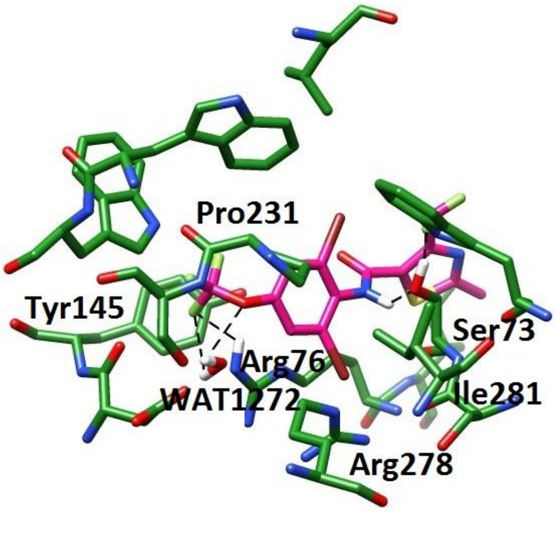

Thifluzamide

Supplementary Figure S6. The binding modes of 18 known SDHIs. The interacting residues are depicted in green stick and compounds in pink color. H-bonds are displayed in black dash lines.

1.1 Supplementary Tables

Supplementary Table S1. Percent identities between *A. solani* SDHs and template sequences

Subunit	Identity	PDB code	Organism	Protein name
**SDH A**	69%	1YQ3	*Gallus gallus*	Succinate dehydrogenase
	69%	1ZOY	*Sus scrofa*	Succinate:ubiquinoneoxidoreductase
	69%	4YTP	*Sus scrofa*	Succinate dehydrogenase
	62%	3VR8	*Ascarissuum*	Mitochondrial quinol-fumarate reductase
	62%	4YSX	Ascaris suum	Succinate dehydrogenase
	55%	1NEK	*Escherichia coli*	Succinate dehydrogenase
**SDH B**	69%	1YQ3	*Gallus gallus*	Succinate dehydrogenase
	67%	3ABV	*Sus scrofa*	Succinate dehydrogenase
	67%	1ZOY	*Sus scrofa*	Succinate:ubiquinoneoxidoreductase
	66%	3VR8	*Ascarissuum*	Quinol-fumarate reductase
	53%	1NEK	*Escherichia coli*	Succinate dehydrogenase
**SDH C**	37%	1ZOY	*Susscrofa*	Succinate:ubiquinoneoxidoreductase
	37%	2H88	*Gallus gallus*	Succinate dehydrogenase
	37%	1YQ3	*Gallus gallus*	Succinate dehydrogenase
	37%	2FLQ	*Geobacillusstearothermophilus*	Nitric Oxide Synthase
	36%	2FBW	*Gallus gallus*	Succinate dehydrogenase
	34%	1MJT	*Staphylococcus aureus*	Nitric-oxide synthase
	33%	2Y5B	*Homo sapiens*	Ubiquitin carboxyl-terminal hydrolase
	33%	3I3T	*Homo sapiens*	Ubiquitin carboxyl-terminal hydrolase
	33%	3MTN	*Homo sapiens*	Ubiquitin carboxyl-terminal hydrolase
	31%	3VR8	*Ascarissuum*	Quinol-fumarate reductase
**SDH D**	42%	2EOB	*Rattusnorvegicus*	1-phosphatidylinositol-4,5-bisphosphate phosphodiesterase
	36%	1ZOY	*Susscrofa*	Succinate:ubiquinoneoxidoreductase
	35%	1YQ3	*Gallus gallus*	Succinate dehydrogenase
	33%	1NP7	*Synechocystissp*	DNA photolyase
	31%	2JFK	*Homo sapiens*	Fatty acid synthase
	31%	2JFD	*Homo sapiens*	Fatty acid synthase
	31%	3HHD	*Homo sapiens*	Fatty acid synthase

Supplementary Table S2. Chemical structure and Docking Scores of 18 known SDHIs (fungicides).

Sr.	Name	Chemical structure	Docking Scores	Sr.	Name	Chemical structure	Docking Scores
1	Benodanil	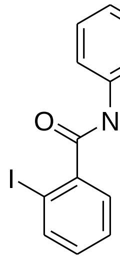	-9.45	2	Benzovindiflupyr	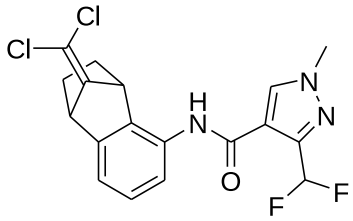	-9.69
3	Bixafen	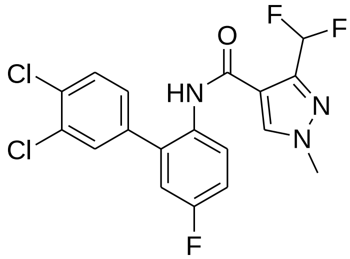	-9.87	4	Boscalid	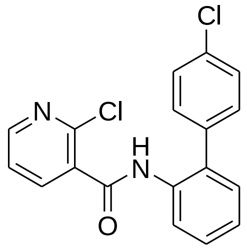	-10.47
5	Carboxin	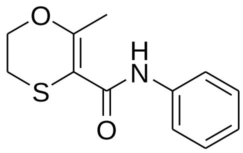	-10.24	6	Fenfuram	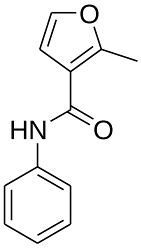	-9.02
7	Fluopyram	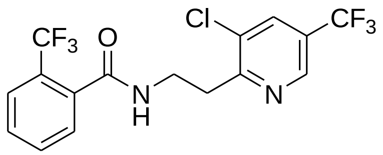	-10.02	8	Flutolanil	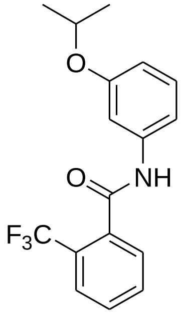	-9.74
9	Fluxapyroxad	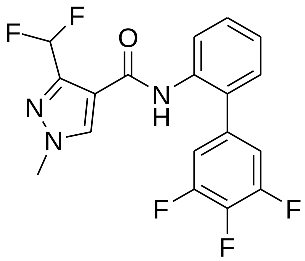	-9.96	10	Furametpyr	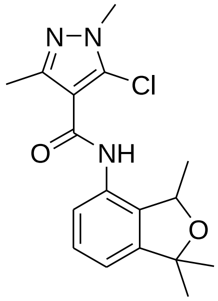	-10.63
11	Isofetamid	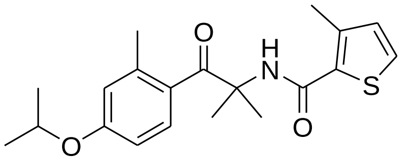	-10.46	12	Isopyrazam	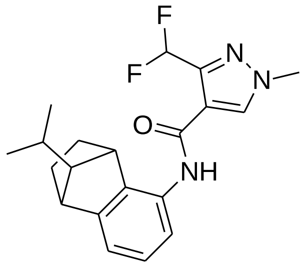	-9.57

13	Mepronil	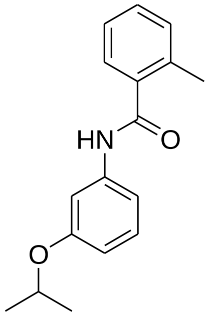	-10.51	14	Oxycarboxin	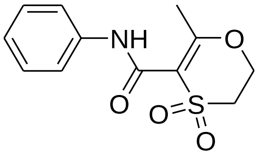	-11.07
15	Penflufen	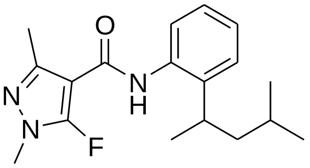	-9.56	16	Penthiopyrad	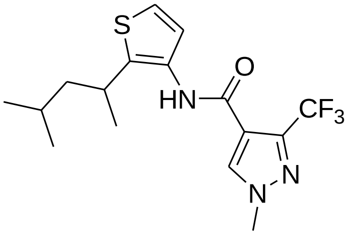	-9.46
17	Sedaxane	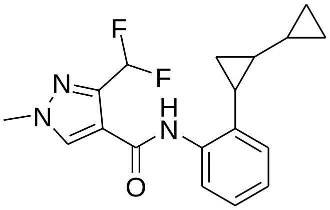	-10.13	18	Thifluzamide	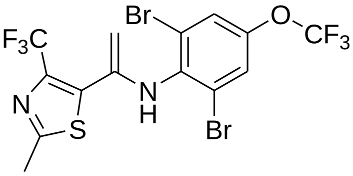	-11.67

Supplementary Table S3. Chemical Structures and Docking Score of 25 compounds selected from ZINC database after docking, rescoring and interaction analysis

No.	ZINC ID	Compound Code	Structure	ADT Score	MOE Score
1	ZINC00781454	**C1**	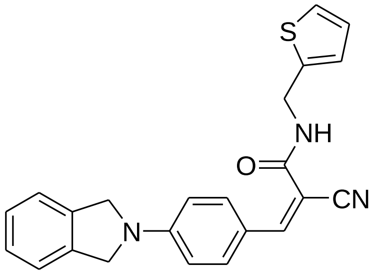	-8.74	-5.2243
2	ZINC13037761	---	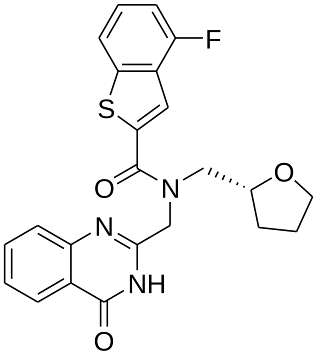	-8.25	-5.7331
3	ZINC78502784	---	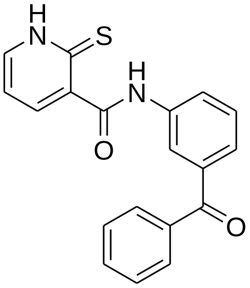	-8.23	-5.2939
4	ZINC19837471	---	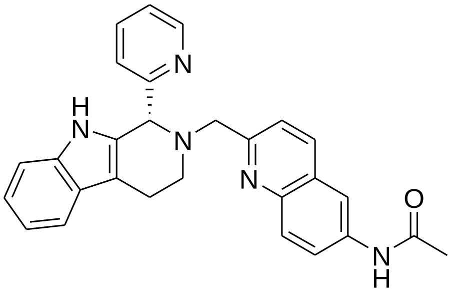	-8.22	-5.2954
5	ZINC50812116	---	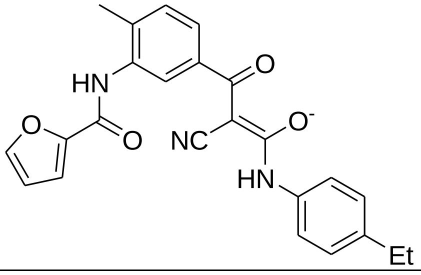	-8.20	-4.4908

6	ZINC04498541	**C2**	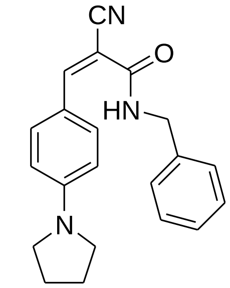	-8.20	-5.2388
7	ZINC34378628	**C12**	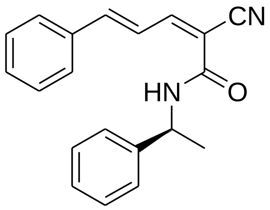	-8.18	-5.1025
8	ZINC27260697	**C3**	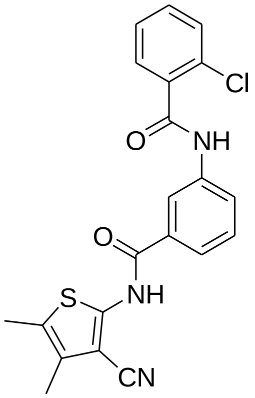	-8.10	-4.1538
9	ZINC00795936	---	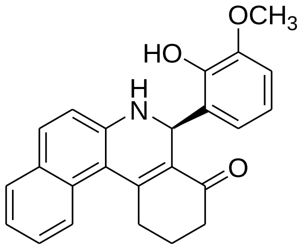	-8.00	-5.2242
10	ZINC32180696	**C11**	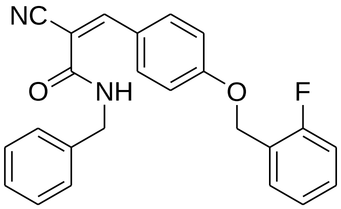	-8.00	-4.6726
11	ZINC36624363	---	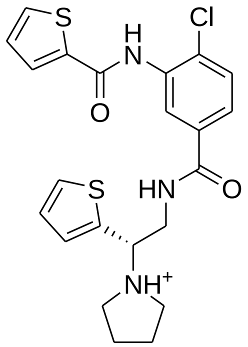	-8.00	-4.6285

12	ZINC17007371	**C10**	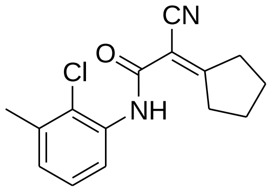	-7.98	-4.4015
13	ZINC06512448	**C9**	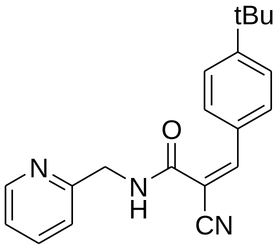	-7.93	-4.8394
14	ZINC04731427	---	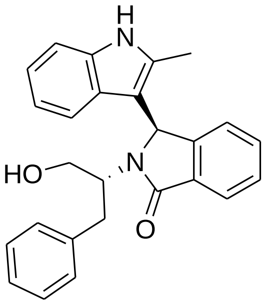	-7.87	-5.0271
15	ZINC32803988	---	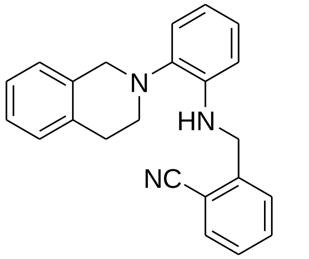	-7.85	-4.3761
16	ZINC02409017	**C4**	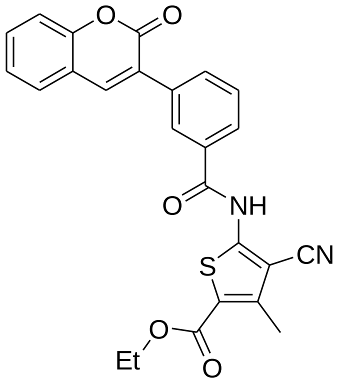	-7.74	-4.5404
17	ZINC03102709	**C6**	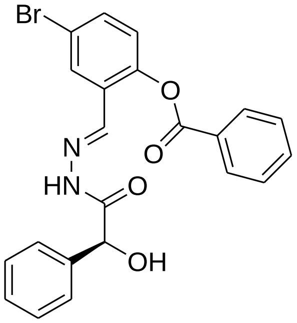	-7.74	- 4.95920

18	ZINC06183077	**C8**	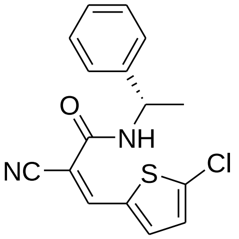	-7.69	-3.4910
19	ZINC00689200	**C5**	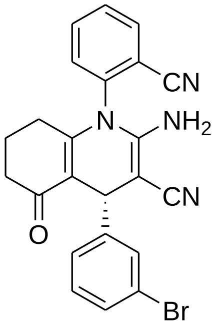	-7.68	-3.7313
20	ZINC01436920	---	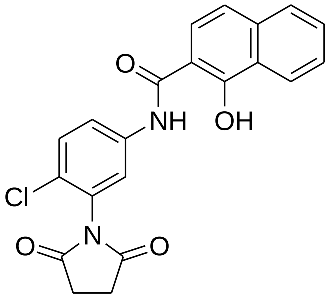	-7.68	-4.9843
21	ZINC18046772	---	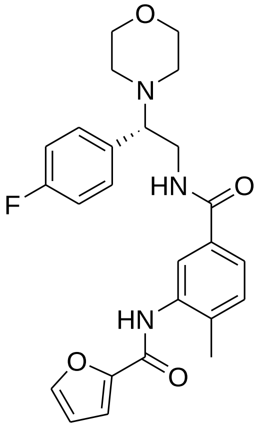	-7.66	-4.4454
22	ZINC04903387	---	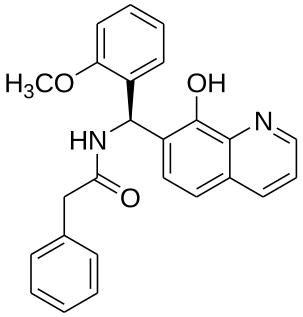	-7.63	-5.2351

23	ZINC05176439	**C7**	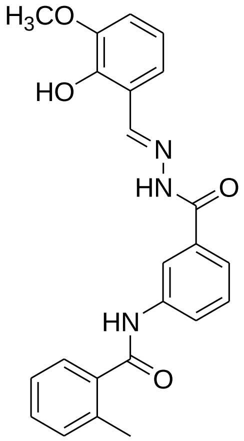	-7.61	-4.3706
24	ZINC00707182	---	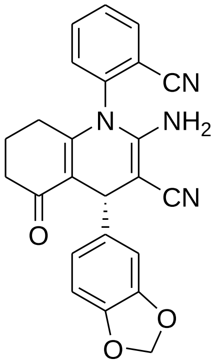	-7.6	-3.5623
25	ZINC12196415	---	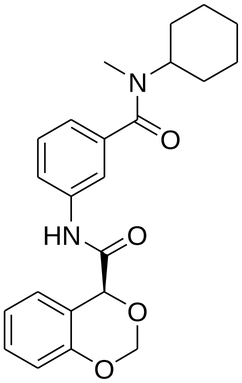	-7.60	-4.3058
